# Effects of straw return and straw biochar on soil properties and crop growth: A review

**DOI:** 10.3389/fpls.2022.986763

**Published:** 2022-09-27

**Authors:** Limei Chen, Songlin Sun, Bin Yao, Yutao Peng, Chongfeng Gao, Tian Qin, Yaoyu Zhou, Chaoran Sun, Wei Quan

**Affiliations:** ^1^ School of Mechanical and Electrical Engineering, Hunan Agricultural University, Changsha, China; ^2^ School of Resources and Environment, Hunan Agricultural University, Changsha, China; ^3^ School of Agriculture, Sun Yat-Sen University, Shenzhen, China

**Keywords:** straw return, agricultural production, soil properties, crop growth, China

## Abstract

Straw return is an effective method for disposing agricultural residues. It not only utilizes agricultural waste but also improves soil. In the current review, different crop straw and its characteristics were highlighted, and patterns of straw return were explored (including straw return, straw biochar return, and their combined with fertilizer return), as well as their environmental impacts were outlined. In addition, the effects of straw return and straw biochar amendment on soil properties [*e.g.*, pH, soil organic carbon (SOC), soil nitrogen (N)/phosphorus (P)/potassium (K), soil enzyme activities, and soil microbes] were discussed. Information collected from this review proposed that straw return and straw biochar return or in combination with fertilizer is an applicable way for improving soil fertility and enhancing crop production. Straw return is beneficial to soil physicochemical properties and soil microbial features. The rice straw has positive impacts on crop growth. However, there are different climate types, soil types and crops in China, meaning that the future research need long-term experiment to assess the complex interactions among straw, soil, and plant eco-systems. Accordingly, this review aims to provide available information on the application of straw return in terms of different patterns of its to justify and to expand their effective promotion.

## 1 Introduction

China is a large agricultural country that has many different types of crop straw, with yields of 797 million tons in 2020 and 802 million tons in 2021 (National Bureau of Statistics data, http://www.stats.gov.cn/); in addition, the average annual growth in the rate of crop residue production was close to 4% (Hong et al., 2016), and straw is a C-rich agricultural waste containing much N, P, K, and micronutrient elements for crop growth (Jin et al., 2020). It plays a very important role in releasing C, N, and P, which can adjust the imbalance of soil nutrients. It has been highly advocated straw resource utilization in China due to the prohibition on the burning straw (Li et al., 2018). Therefore, crop straw should be seriously considered as a resource to make, full use of agricultural residues and protect the environment.

At present, soil degradation, SOC and nutrient loss, as well as reductions in soil fertility, are the primary problems faced by Chinese agricultural development. Healthy soil is important for the crop growth, thus protecting human health (Yu et al., 2019). In recent years, with the continuous enhancement in crop yields, crop straw yields have also increased. Rice straw, corn straw and wheat straw account for 90% of the total straw production in China (Ma et al., 2020). Many scholars have carried out extensive research on soil health management. Crop straw itself comes from farmland; thus, returning it to farmland is a good agricultural management strategy. Straw return can effectively increase the soil aggregate structure and improve soil properties (Jin et al., 2020; Hu et al., 2021). However, the traditional method of directly returning crop straw to the field has many problems in terms of the utilization of straw resources. For example, straw mulching and shallow plough are mostly used in the field, usually resulting in slow straw decomposition, an inability to quickly absorb and utilize crop straw, poor soil organic matter improvement effects, and reduced crop yields (Dong et al., 2018; Hu et al., 2021). On the other hand, crop straw is bulky, resulting in high recycling costs; thus, many farmers choose to burn crop straw directly, which exacerbates the greenhouse effect. Currently, the preparation of straw biochar by pyrolyzing organic materials from agricultural wastes such as crop stalks has attracted increasing attention worldwide (Semida et al., 2019; Gong et al., 2022). Straw biochar has great potential for use in soil C sequestration and soil fertilizers, and it could be used as a C-based fertilizer or in combined with organic/inorganic fertilizers application. Therefore, different straw return patterns on soil are worth an in-depth discussion in relation to changes in soil properties and influences on crop growth.

Returning crop straw to the field is a sustainable solution to boost soil fertility and promote crop growth. Nevertheless, a recent compilation of information on the overall impact of different straw return patterns on crop productivity and soil fertility showed this information is limited. Hence, there is an urgent need to synthesize the above information. There exists complete understanding on straw management practices and crop straw efficiently in China. This article summarized the nutrient characteristics of different crop straws and their role in soil fertility, which affects crop growth. Thus, the purposes of this study were to investigate (i) crop straw characteristics from different materials, (ii) the responses of straw or straw biochar or the co-application of fertilizer and straw biochar on soil properties and crop growth, as well as (iii) propose future perspectives and directions. We believe that this article will greatly help in planning reasonable straw return models.

## 2 Methodology

**Figure 1:**
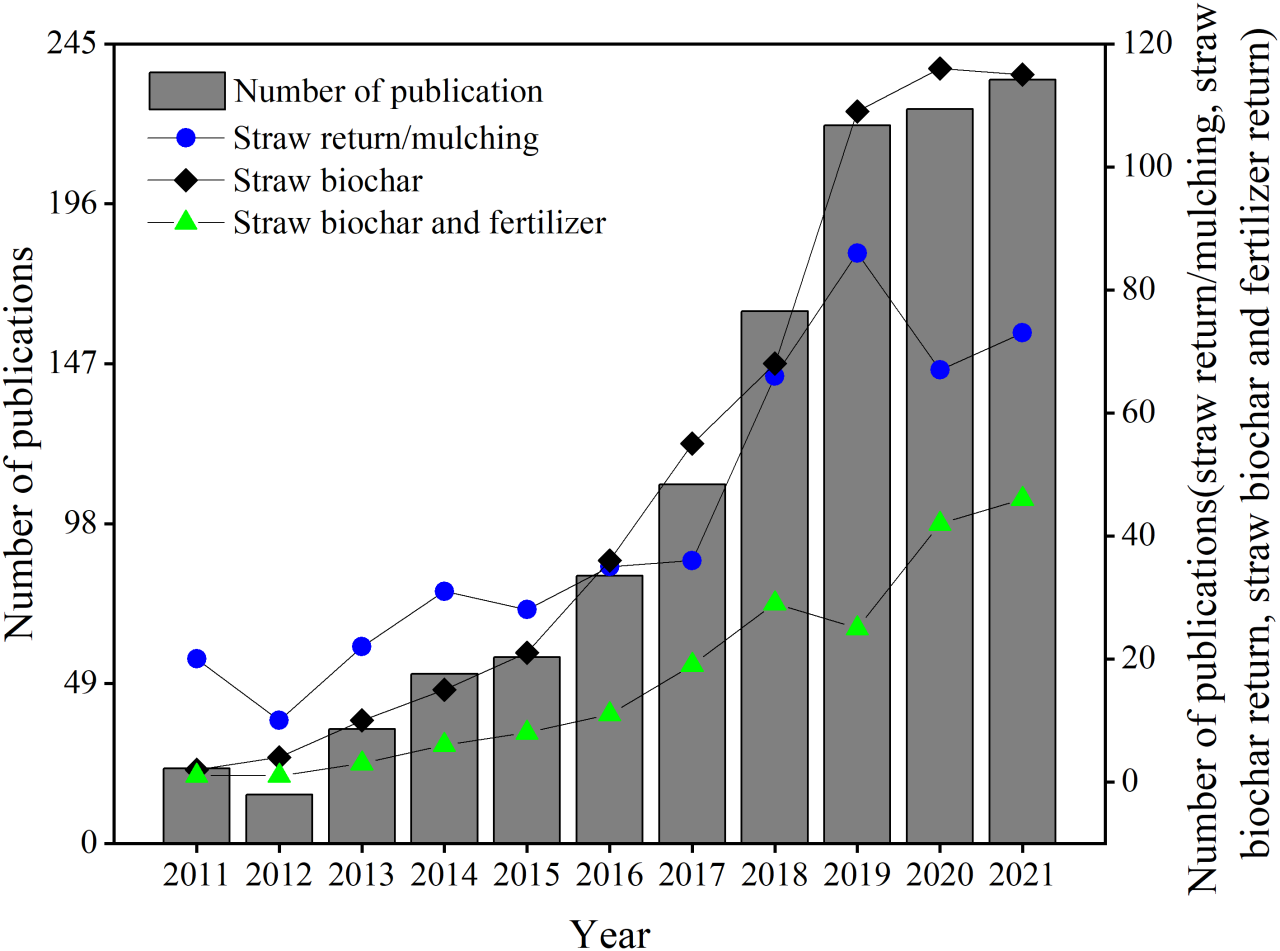
Publication performance during 2011 to 2021.

Google Scholar and Web of Science were used to search keywords, such as “straw return”, “straw biochar return”, “fertilizer and straw biochar”, “soil properties”, and “crop growth”. Additional papers were searched using the keywords “straw mulching”, “stalk return”, “ straw-derived biochar”, “straw biochar”, and “crop straw incorporation”. Only the relevant papers that matched these keywords were selected as the foundation of this review. The time period of 2011 to 2021 was prioritized. The searched reference results related to this paper are shown in **Figure 1**, including those related to straw return/mulching, straw biochar return, combined straw biochar and fertilizer return. The numbers of publications for each year are depicted in **Figure 1**. The number of publications has been growing over the past ten years (**Figure 1**), showing that research on straw return is becoming an increasing focus in China. Crop straws are rich in nutrient resources such as C, N, P, and K, and the contents of these nutrients in straw are 36.6–50.36%, 0.33–2.28%, 0.05–0.45% and 0.23–2.45%, respectively (**Table S1**).

## 3 Influence of directly returning straw on soil quality and crop production

### 3.1 Soil quality

**Figure 2:**
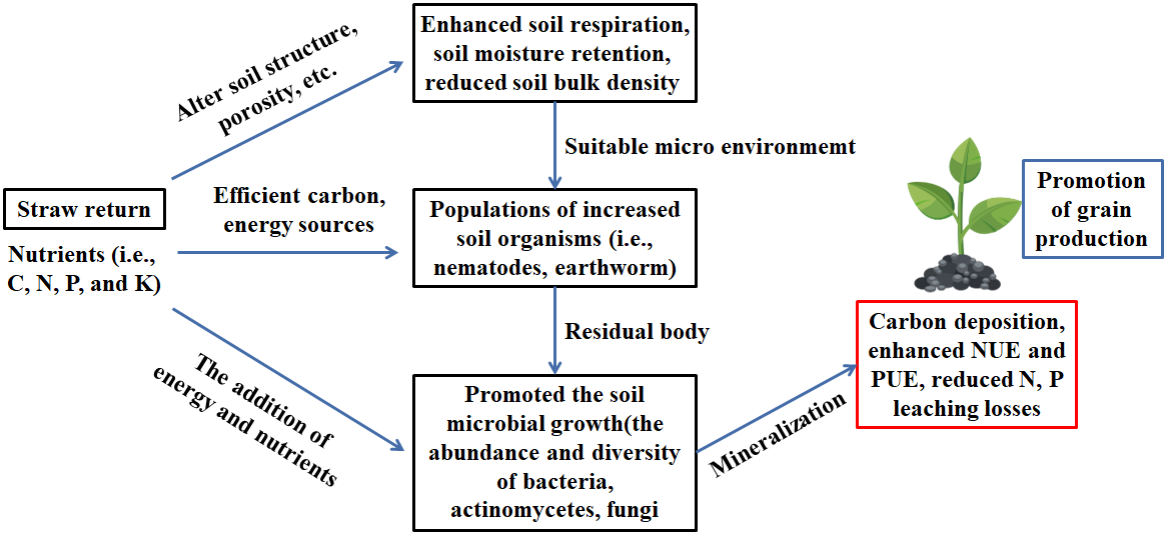
Straw return impact in soil for crop growth.

The application of crop straw return and incorporation into soil as independent treatments could affect soil physicochemical properties, including soil pH, SOC, soil N, P, K, etc. In addition, returning straw to field could also improve soil biological characteristics by enhancing diversity and supplying szhang uitable conditions for soil microbial communities (Li et al., 2019b). Straw return is a good management for regulating soil nutrients and decreasing soil C, N, P and K losses in farmland. Thus, straw return could improve soil properties by changing their effects mechanisms, such as increasing soil respiration, soil organisms and soil microbial growth (**Figure 2**). Crop straw properties support its long-term agronomic and environmental benefits. Crop straw is utilized in various ways in agricultural management, primarily by being directly applied the soil, returned to the field as straw biochar, or combined with fertilizer (He et al., 2015; Cong et al., 2020). Different research data for China in recent years are shown in **Table S2**. Compiled information includes straw feedstock, crop types, soil type, and primary influences of straw return addition on soil parameters. In summary, previous studies have shown that soil properties in straw return-amended soils depend on the crop-straw application rate, type of soil, kind of crop, and depth of the soil layer (**Table S2**).

#### 3.1.1 Soil pH and soil organic carbon

The growth and development of crops are severely affected by soil pH. Soil pH mainly affects the growth of crops by changing the physical, chemical, and biological soil characteristics, thus affecting the growth morphology, quality, and yield of crops. The increase or decrease soil pH by returning crop straw to farms is affected by straw feedstocks, different application levels and soil classes (**Table S2**). Overall, the straw that is returned to the field has little effect on soil pH.

Current studies have indicated that returned crop straw could promote SOC stocks (Chen et al., 2015; Zhao et al., 2018). Returning crop straw and fertilizers to the field also significantly increases the content of SOC (**Table S2**), which may be due to the relatively high C contents of crop straw. However, in comparison to fertilizer application alone, crop straw application has been shown to result in lower SOC, while a combined application of the two had a better effect (Bai et al., 2021). For instance, Zhang et al., (2016c) reported that in comparison to that of compost plus 70% NPK fertilizer, the utilization of straw and compost plus 70% NPK fertilizer increased the SOC by 8.59%, 0.90%, and 8.40% in 2012, 2013, and 2014, respectively. Zhang et al. (2016b) also indicated that a NPK fertilizer plus wheat/maize straw addition resulted in 26–38% higher SOC compared to that with no fertilizer and no straw return from an experiment. Another study showed that in contrast to the original soil, mineral fertilizer and straw return significantly increased SOC storage by 7.19%, and the SOC storage markedly decreased by 3.47% in the no fertilizer treatment after 13 years (Hao et al., 2020). At the same, returning straw is useful for C sequestration, mainly due to the increased humic acid C after straw return (Hao et al., 2020). Straw contains a variety of nutrient elements that can be used as soil microorganisms; however, this depends on the application amount of crop straw in the field, which needs to be determined according to the requirements of the crop. For example, Zhu et al. (2015) found that compared to the 0% straw return treatment, the straw return amendment (25%, 50%, 75%, and 100%) primarily increased the SOC and labile organic C fraction contents at 0–21 cm soil layer. Apart from the 100% straw return treatment, the dissolved organic C content of all straw return treatments was significantly higher than that of the 0% straw treatment in the 0–21 cm soil layer. Additionally, returning 50% of the straw to the field every year was the best choice for improving SOC sequestration. Conversely, with increasing straw return rates (0%, 25%, 50%, and 100%), the C storage influences of the N-treated and NPK-treated topsoil (0–21 cm) become obvious (Lou et al., 2011). Moreover, many studies have reported long-term experimental study-induced changes in SOC. For instance, Li et al. (2019a) revealed that compared to the initial value, the SOC reserve increased by 16.4% over a 6-year period when the wheat straw and corn straw were returned (Li et al., 2019a). A long-term field experiment (1985–2017) was set up with different tillage patterns, different application ratios of mineral fertilizers and crop straw on the North China Plain. According to this experiment indicated that the SOC content increased rapidly over the past 15 years because these farmlands where wasteland before the treatments were implemented (Zhang et al., 2021). In addition, different tillage methods also affect SOC content. Liu et al. (2015) pointed out that straw return to the field or no-tillage, especially the combined with straw and no-tillage, could effectively reduce soil erosion and C sequestration in dryland agriculture in Northern China. Study of Fan et al. (2020) shown the mixed soil with straw return was able to markedly increase SOC content. At the same time, they found that straw mulching and straw crushing significantly promoted the formation of soil macro aggregation, increasing the related C content of soil surface macro aggregation by 23.69% and 21.70%, respectively. Besides this, the SOC content of the straw return treatment was generally higher than that of the straw removal treatment (Yang et al., 2015), and straw return to the field has great potential to increase SOC (**Table S2**). In general, crop straw return to the field could increase SOC inputs and benefit the local environment.

#### 3.1.2 Soil nitrogen, phosphorus, and potassium

Application crop straw influences soil N, P, and K contents. The changes in N, P, and K as affected by different crop straw types are indicated in **Table S2**. Straw return is a valid way to reduce soil N, P, and K loss, but the benefits differ based on soil texture. Based on the research, when crop straw was added to soil, total N, ${\text{NH}}_{4}^{+}{\text{-N, NO}}_{3}^{-}\text{N}$ , available N, total P, available P, total K, available K, etc. were found to be stimulated (**Table S2**). According to the research for a field microplot experiment, straw return improved the N content and N use efficiency. The contents of ${\text{NH}}_{4}^{+}\text{-N}$ increased and ${\text{NO}}_{3}^{-}\text{-N}$ decreased in straw mixed or buried at depths of 0–20 cm (Tian et al., 2020). Zhang et al. (2019) predicted that residual N from peanut straw can replace wheat growth with 104,500 tons of synthetic N fertilizer every year because the peanut straw return retained a large amount of N produced by the decomposition of plant rhizosphere microorganisms. Increasing straw return to the field by 26.4% can reduce N fertilization by 8 kg·ha^-1^·year^-1^ and improve the efficiency of fertilization (Liu et al., 2018). Returning straw to the field also reduces N loss by promoting soil structure, thereby increasing water infiltration (Xia et al., 2018), and improving the N content in the soil. In addition, more SOC content after straw return increases the cation exchange capacity to prevent the loss of ${\text{NH}}_{4}^{+}$ and enhances the ability to maintain the moving anion ${\text{NO}}_{3}^{-}$ because of the deprotonated carboxyl group. Therefore, straw returned to soil can boost soil N content (**Table S2**).

Previous studies indicated that the crop straw return can provide total K_2_O, most P_2_O_5_, and a part of the N content (Yin et al., 2018). Returning straw to the field has the potential to reduce fertilizer use and the environmental burden. Soil P availability has an important influence on crop growth. Returning straw to the field can increase the P content of the soil, which may be because inorganic P is transformed into organic P after vast amounts of straw are returned to the field, thereby maintaining the relative stability of the soil organic P pool. Nevertheless, the long-term continuous addition of straw with K fertilizer to the field significantly reduced the K fixation capacity of the soil (Tan et al., 2017). In future research, straw mulching should be used as the optimized K supply to crops. A 60-day laboratory incubation experiment by Li et al. (2020) confirmed that the content of K was retained when straw return was combined with K fertilizer, the available K increased by 72.9%, and the wheat and maize straw use rates increased by of 47.1% and 39.3%, respectively. As shown in **Figure 2**, there is increasing evidence that returning straw to the field improves soil fertility and promotes grain production, although some negative effects have been reported.

#### 3.1.3 Soil enzyme activities and microbes

Straw return can promote soil microbial activity and thus has an important regulatory effect on soil enzyme activity. Soil enzymes are indicators of soil health and play an important role in the soil nutrient cycle of the agricultural ecological environment (Zhao et al., 2016). The application of the maize straw and NP fertilizer increased the *β*-glucosidase, *β*-xylosidase, and N-acetyl-glycosaminidase activities, compared to the CK (no-fertilizer) treatment (Zhao et al., 2016). Simultaneously, compared with the NP fertilizer treatment, all the enzyme activities increased by 10.5–32.8% with the addition of maize straw and NP fertilizer. However, some research results are not the same (**Table S2**). Previous studies have reported that when straw was applied, the soil urease and acid phosphatase activities in the 0–20 cm soil layer were higher, while straw that was returned and was ploughed both increased the soil acid phosphatase activity in the 30–40 cm soil layer (Tian et al., 2020). Mixing straw into deep soil for ploughing tillage may accelerate nutrient cycling in the subsoil layer, thereby increasing soil enzyme activity. However, another study showed that returning straw to the field with rotary tillage significantly increased the soil enzyme activity at 0–10 cm (Chen et al., 2017). More importantly, the activity of soil enzymes can be improved by increasing the secretion of soil enzymes, thereby moderately changing the soil community.

Soil microorganisms, which mainly include bacteria, actinomycetes and fungi, are the most important indicators of soil quality. While, the straw return normally provide efficient C and energy sources for soil microorganisms. Recent research proved that straw return affects soil bacterial communities from a field experiment. Resulted showed that the interaction of stalk plus elevated CO_2_ significantly affected the abundances of 10 genera. Moreover, different genes had different abundances under straw return (Miao et al., 2021). Upon the straw return, the abundance of bacteria and fungi 1.4 times and 4.9 times, respectively, indicating that straw return resulted in fungi being the dominant flora in the soil (Xiao et al., 2020). In contrast, Yang et al. (2019) observed that wheat straw addition had no effect on the soil fungal community, but it changed the α-diversity of soil bacteria. At the same time, the bacterial abundance, richness, or diversity in the 0–45 cm soil layers were not affected by straw return (Yu et al., 2018a). As a result, returning straw to the field is beneficial to soil structure, affecting microbial activity and most likely soil nutrient cycling, especially increasing SOC reserves.

### 3.2 Crop production

Straw return has been inspected as an environment-kindly model for the straw application owing to its beneficial influences on the soil fertility and the crop production. Therefore, crop straw return is widely applied in the agricultural field. For example, straw incorporation into the field (mixed or chopped) resulted in higher annual wheat and corn yields than that in the control treatment in an eight-year study (Zhao et al., 2019). Soil quality and crop yield will be affected by different tillage methods and straw management methods. Another study reported that wheat stalks return and moldboard plough tillage boosted soil fertility by changing crop nutrient absorption, utilization, and transfer to affect crop growth (Zhao et al., 2021). Thus, the accumulation of peanut dry matter and N promoted an increase in peanut yield. Some field trials have shown that ditch-buried straw in the field supplied a basis for crop maintenance and even increased production. In this trial, two crop yields and their components were significantly increased at 0.5 t·ha^-1^ straw buried at 35 cm (Yang et al., 2016). A 5-year field experiment also found that returning corn straw every year and deep ploughing every 2 years could promote the root length density in the 10–40 cm soil layer and increase the grain yield (Chen et al., 2020). In addition, Wang et al. (2019) found that ploughing is a more suitable method of straw return (3.2 t·ha^-1^) than straw mulching in the rape-rice rotation pattern. According to a new planting pattern known as “broadcast sowing with straw return” has been adopted, grain yield improved without decreasing grains per spike and the 1000-grain weight (Zhai et al., 2018). Furthermore, straw return decreased the impact of higher grain yield by the co-application of mineral fertilizer or NPK fertilizer (**Table S3**). It can be concluded from **Table S3** that returning straw alone and returning straw to the field together with other fertilizers can almost increase crop productivity. Another study also showed that straw return in combination with a low-N environment appropriately stimulated deep roots, thereby increasing grain yield (Xu et al., 2018).

## 4 Effect of straw biochar return on soil quality and crop production

Crop straw returned to the soil decomposes slowly since crop straw has high lignin and cellulose contents and thus affecting crop production. Therefore, straw or straw biochar return have high research value for crop growth and the implementation of sustainable agriculture and environmental protection. Biochar can promote plant production by improving soil properties (pH, SOC, nutrient retention and availability, etc.). Additionally, biochar improves the biological characteristics of soil by altering the structure of the microbial community to provide a suitable environment for the soil microbial community (Nielsen et al., 2018). Biochar as a soil modification helps to enhance the properties of degraded soils as biochar is enriched in the nutrient elements C, N, P, K, etc. (**Table S4**). Among them, C content accounts for more than 40%, straw biochar composition depends on pristine materials and carbonization temperature, and many articles expressed that there are differences in the composition of straw biochar (**Table S4**).

### 4.1 Soil quality

#### 4.1.1 Soil pH and soil organic carbon

Straw biochar has the capacity to change soil structure and soil quality due to its porous structure and large specific surface area. Nevertheless, different types of straw biochar have different effects on different soil types and their crop effects. Straw biochar is mostly an alkaline material, which is mainly affected by some inorganic minerals (*e.g*., carbonates and phosphates) and the ash generated during the carbonization process. In addition, since the organic acids of biomass will volatilize as the temperature increases during the pyrolysis process, the pH of straw biochar will increase with increasing carbonization temperature (Chen et al., 2019). The effect of adding straw biochar on soil pH is generalized in **Table S4**. Straw biochar could enhance or decrease soil pH in different soil types. The reason for this situation may depend on the types of soil, crops, and straw biochar, among others.

At present, some studies have revealed that the application of straw biochar can increase the SOC content (**Table S4**). Zhang et al. (2018) reported that in comparison to the control treatment rice straw addition, amended straw biochar with rice straw addition, and straw biochar application significantly increased the variety of organic C contents (such as readily oxidizable organic C, dissolved organic C, and light fraction organic C). In addition, they set up 7 application rates at 0, 2.5, 5, 10, 20, 30, and 40 t·ha^-1^ in a field experiment and observed that the 40 t·ha^-1^ straw biochar-treated increased mineralization by approximately 4.62–6.91%, and it decreased over the year. Straw biochar is rich in C, and the C contents of the straw in **Table S4** is as high as 46.72–88.82%. Thus, returning straw biochar to the field is an important C sequestration measure for agroecosystems.

#### 4.1.2 Soil nitrogen, phosphorus, and potassium

Straw biochar amendment has a strong adsorption capacity and is a valid means for improving the effectiveness of soil nutrients. Straw biochar can participate in the nutrient cycling process by interacting with soil, such as adsorbing various nutrient elements on the surface (*i.e.*, N, P, and K) and enabling ion exchange. Most previous studies have demonstrated that straw biochar amendment could increase crop N, P, and K concentrations, which is summarized in **Table S4**. Of course, reports on straw biochar on soil N, P, and K mineralization do not have consistent results when applied to different soil types (**Table S4**), and the results include reduced and increased mineralization and no impact. Furthermore, studies have shown that the impact of straw biochar on soil nutrient contents is affected by the type of crop and crop straw. Straw biochar application is also affected by the ratio of biochar applied. Uzoma et al. (2011) demonstrated that soil total N, available P, and exchangeable K contents increased with the straw biochar application ratio. In another study, the available N, P, and K in the soil differed with the amount of straw biochar used, and the soil available nutrients may increase with straw biochar application rates but may also decrease at high application rates (60 t·hm^-1^) (Li et al., 2018). Straw biochar amendments can improve soil fertility because they contribute to the biochemical cycle of N and P. The C content of straw biochar increases significantly as the carbonization temperature increase, and the C content of corn straw biochar is the highest, as shown in **Table S4**.

According to the lack of nutrient elements in the soil, straw biochar can meet the needs of crops for specific nutrient elements. The beneficial effect can be attributed to the inherent nutrients in straw biochar, and their availability depends on the nutrient elements of straw biochar. Most straw biochar contains C, N, P, and K, and the addition of straw biochar can almost meet the K content of crops without applying conventional K fertilizer (Wang et al., 2018). This scenario occurs because K is largely preserved during the pyrolysis process and converted into a K-containing salt with high solubility (Karim et al., 2017). Therefore, the high available K content in straw biochar is the reason for the increasing soil potassium concentration. Similarly, straw biochar has significant available N and available P; thus, available nutrients are diametrically incorporated in soil by straw biochar application. Also, alkaline straw biochar can form new functional groups by increasing soil pH and soil cation exchange capacity to improve soil available nutrients, which is indirect evidence of nutrient richness due to the addition of straw biochar to the soil.

#### 4.1.3 Soil enzyme activities and microbes

Straw biochar application amount and soil type affect changes in soil enzyme activity. Soil enzymes are major parameters of soil quality because they can affect many soil microorganisms and their community structure and richness. For example, long-term laboratory research showed that enhanced maize straw biochar amendment resulted in notably increased SOC, total N, and available K, while the performance of soil enzyme activity was the opposite. A small maize biochar addition (0.5%[*w*/*w*]) accelerated soil enzyme activities, whereas soil enzyme activities were reduced at higher levels of biochar addition (1.0, 2.5, and 5.0%[*w*/*w*]) (Wang et al., 2015a). Furthermore, Song et al. (2018) found that soil enzymes related to C cycling were enhanced by the application of biochar; compared with the NPK treatment, the corn straw biochar prepared at a pyrolysis temperature of 600°C did not affect the soil enzyme activity, while the biochar produced by pyrolysis at 300°C and 450°C increased the enzyme activity related to soil C and N conversion. The straw biochar produced by pyrolysis at 450 and 600°C promoted first an increase and then a decrease in the enzyme activity related to C conversion. Among the enzymes, soil urease activity experienced minimal effects (Wang et al., 2015b). This result showed that the temperature of straw biochar pyrolysis also affects soil enzyme activity. Hence, the current research on the regulatory mechanisms of different pyrolysis temperature of straw on soil enzyme activity needs to be expanded.

Straw biochar application have a role in adjusting enzyme activity through soil microbial activity improvement. Dissolved organic C content affects the diversity of soil microbes. For instance, studies showed that compared with returning rice straw to the field, the incorporation of rice straw and biochar sharply increased the abundance of bacteria and total microorganisms. This is because the addition of rice straw biochar increases rather than the addition of straw, which improves soil microbial C metabolism and diversity (Zhang et al., 2018). Therefore, the release of soluble organic C in the soil leads to the enhancement of soil microbial C metabolism and diversity by rice straw biochar. The impact of straw biochar on microbial communities mainly depends on the application rate of straw biochar and the type of soil. A short-term incubation experiment found that straw biochar amendment resulted in high relative abundances of gram-negative bacteria in paddy soil. Straw biochar had a weak effect on microbial biomass, and there was a positive correlation between the relative abundance of the microbial community and C availability (Pan et al., 2016). Straw biochar application can stimulate soil microbial activity and diversity, and straw biochar application rates significantly affect soil moisture retention capacity and soil pH and increase soil nutrient retention, changing soil microbial functions and community structure (Palansooriya et al., 2019). Similarly, high rates of straw biochar amendment could change soil microbial communities. For example, the addition of maize straw-derived biochar at 30 t·ha^-1^ promoted the percentage of fungi, but fungi and bacteria ratios were obviously higher in the 50 t·ha^-1^ biochar application; thus, the soil fungi increased with high amounts of biochar. Besides, the biochar level at 30 t·ha^-1^ increased SOC, total N, and total P; thus, an increase in the relative abundance of fungi was observed when compared to that in the control (Luo et al., 2017). More importantly, the changes in straw biochar application and farming system that improve bacterial and fungal community structure in the soil need to be further studied.

### 4.2 Crop production

**Figure 3:**
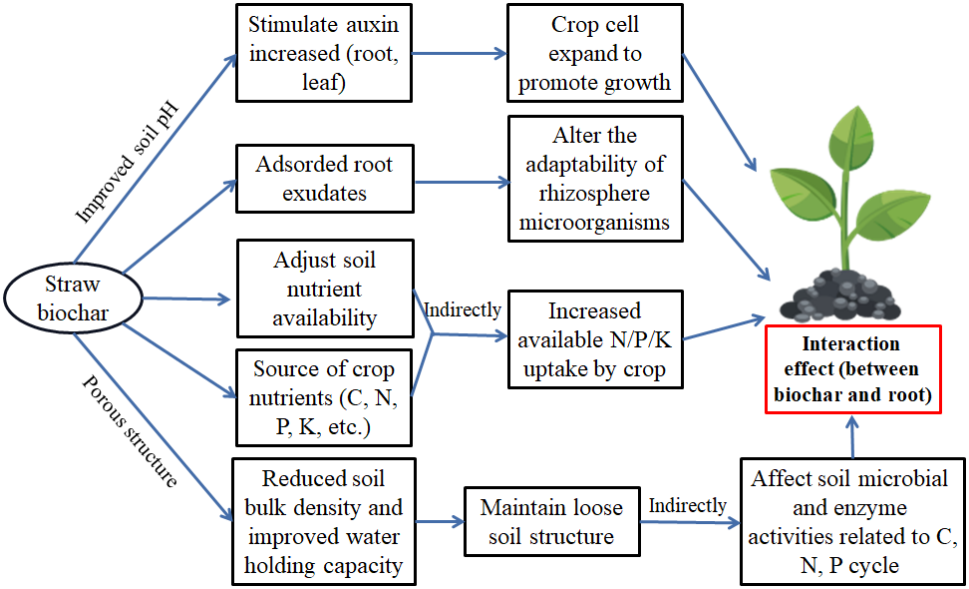
Straw biochar return impact in soil for crop growth.

Straw biochar has been proven to have application value for improving soil quality and increasing crop productivity. The possible influence mechanism of straw biochar amendment on soil quality and crop growth is shown in **Figure 3**. **Table S5** provides a detailed overview of the impact of straw biochar in different soil types and different crops on plant productivity. Many studies have different results for crop yield and crop growth. These divergences seem possible to depend on the straw feedstock, soil types, climate conditions, and their treatments (**Table S5**). On the other hand, a recent meta-analysis indicated that biochar submissions could heighten above-ground plant production by –10% or even –25% (Liu et al., 2013). Huang et al. (2019) found that the use of straw biochar resulted in a decline in rice yields in the first three seasons, but due to the increase in sink size and total biomass, grain yields also increased by 4–10%. Additionally, the rice yield and yield attributes had positive effects on the stage of straw biochar addition. While straw biochar application also has the opposite results. A field experiment investigated that the addition of straw biochar (10, 20, 40, and 60 t·hm^-1^) slightly stimulated plant height (Li et al., 2018), but this effect was not significant. This result implies that straw biochar does not promote crop growth overall when amended with high amounts of straw biochar. An experiment from 2011 to 2018 showed that grain yield decreased with excessive straw biochar application (11.25 Mg·hm^-1^), while a low dose of straw biochar resulted in the highest yield (Xie et al., 2021). This result shows that the application of high amounts of straw biochar may inhibit crop productivity.

## 5 Impact of fertilizer and straw biochar return on soil quality and crop production

### 5.1 Soil quality

**Figure 4:**
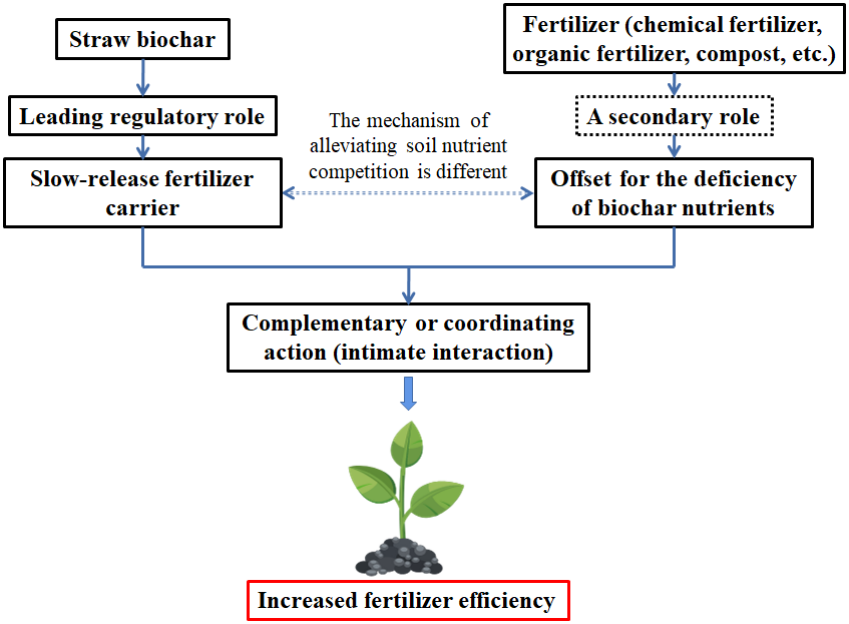
Straw biochar and fertilizer impact in soil for crop growth.

Most papers provided results related to the straw biochar impacts to soil properties and crop growth. In addition, many researchers examined the effects of the co-application of straw biochar with fertilizer to soil. In contrast to the addition of straw biochar alone, straw biochar and fertilizer could provide more C sources and N content, which benefit crop growth (**Figure 4**). Several studies have reported that straw biochar has a large effect on soil properties, such as soil pH, SOC, total N, total P, available N, available K, and microbial biomass (**Table S6**). The varies in soil properties as affected by different crop straw biochar types are revealed in **Table S6**.

#### 5.1.1 Soil pH and soil organic carbon

Generally, the application of fertilizer and biochar increase soil pH (**Table S6**). As a good soil additive, straw biochar can provide alkaline cations (K, Ca, and Mg) (Zhang et al., 2016a). Besides, moderate damage to acidic functional groups has been shown with the application of straw biochar (Mukherjee et al., 2011). On the other hand, straw biochar application increase soil pH due to its liming effect on soil. Its strong alkalinity allows straw biochar to ameliorate soil acidity (pH>7, **Table S6**). As shown in **Table S6**, straw biochar is alkaline. Thus, straw biochar utilization could increase soil pH. Yu et al. (2018b) manifested that when the acidic soil pH was improved, there was a beneficial environment for sustaining microbial reproduction and development. Wang et al. (2017) also revealed that peanut shell composted biochar-based amendments increased soil pH and soil N contents. The reason for the increase in soil pH is the OH^-^ and ${\text{HCO}}_{3}^{-}$ emissions and the ${\text{NO}}_{3}^{-}\text{-N}$ increases.

According to reports, to improve soil fertility, part of the inert C of straw biochar directly increase SOC, while the other part of the available C is used by soil microorganisms to increase microbial C (Liu et al., 2021). According to the research for an experiment, the SOC increase with the addition of biochar every year, showing that straw biochar carrying inert C increases the SOC content (Liu et al., 2021). An increased in SOC by straw biochar addition can reduce the mineralization of background SOC by enhancing the interaction between organic minerals (Weng et al., 2017) and increasing the structure and stabilization of soil aggregates at 250–2000 μm, which promotes SOC to decrease C loss (Zhang et al., 2020). However, in the noted study, the SOC content was strikingly higher when straw biochar was combined with the fertilizer (*i.e.*, manure, NPK fertilizer, and organic fertilizer) treatment compared to the single straw biochar or fertilizer treatment. Tang et al. (2020) reported that combined application of straw biochar with compost promoted soil pH and SOC content compared to the separate addition of straw biochar or compost. The addition of fertilizer to the soil also provided some benefits for increasing SOC, especially organic fertilizer. Organic fertilizer could increase the stock of soil organic matter; thus, clearly, organic fertilizer amendment is better and crucial for nutrient preservation and nutrient availability. It is particularly notable that the interaction of straw biochar and organic fertilizer factors on mineral-associated organic C was significant, thereby increasing SOC (Plaza et al., 2016).

#### 5.1.2 Soil nitrogen, phosphorus, and potassium

Previous studies have reported that, combination of straw biochar and fertilizer could be considered a low-release fertilizer that increases soil quality by increasing the soil total N, total P, total K, and available N/P/K contents. According to Jin et al. (2019), after applying corn straw biochar with NPK fertilizer, soil nutrients increased, and the soil C/N ratio also improved. The combination of straw biochar and fertilizer may have played a role in nutrient maintenance and soil fertility enhancement. At the same time, in this study, the co-application of straw biochar and fertilizer increased the N contents due to their synergistic effect promoting soil organic N mineralization and reducing N leaching (Yu et al., 2016). These increases could have been due to the unstable C, N, P, and K contained in the straw biochar and the consecutive release of these nutrients into the soil. In addition, organic fertilizer (compost, manure, etc.) is a rich source of crop growth. Therefore, the straw biochar combined with organic fertilizers has proven to be a great method for improving soil quality and crop production. A four-year field study showed that compared to the control, the straw biochar and compost application treatment resulted in 300% higher available P contents during the first three years (Safaei Khorram et al., 2019). An association of compost with straw biochar helped to improve the use efficiency of N/P/K in the soil. A similar behaviour was observed in that the available nutrients in the soil improved after chicken manure and straw biochar amendments (Liu et al., 2020).

#### 5.1.3 Soil enzyme activities and microbes

Most variations in soil qualities are related to biological factors, including microbial biomass, microbial communities, and enzyme activities. Many studies have shown that the use of straw biochar generally promotes soil enzyme activities associated with C, N, and P cycling; however, different studies have obtained different results, which indicates that straw biochar has inconsistent effects on different soils and enzymes. Results from a redundancy analysis and found that the alterations in soil microbial community structures were mainly affected by the contents of microbial biomass C, microbial biomass N, available K, and the C/N rate (Song et al., 2018). Nevertheless, straw biochar amendments are not always positive. Tang et al. (2020) showed that a compost treatment could modify the activities of soil enzymes; however, the soil enzyme activities were inhibited by the application of straw biochar, while the co-application of biochar and compost exhibited highly variable responses.

Straw biochar involves a large amount of C, N, P, and K and thus can directly lend nutrients to the soil and can also be used as a nutrient source for microorganisms. According to the results of a study, compared to an NPK-amended soil, the addition of NPK and maize straw biochar to soil significantly affected the soil microbial biomass C and microbial biomass N by 20.68–46.00% and 189.95–233.11%, respectively. At the same time, compared with the NPK treatments, all the NPK with rice straw biochar-applied treatments impressively improved the total PLFA contents by 6.73%–12.07% (Song et al., 2018). Similar or opposite conclusions have been obtained in previous field studies; straw biochar application to soil has been shown to increase or decrease soil community structure and abundance, depending on the difference in straw biochar and microbial communities. However, a few recent studies have found that straw biochar has nonsignificant effects on soil microbial community. For example, a study was executed in tubs to determine the influence of straw biochar with N fertilizer in the rhizosphere of soybean. The results showed that in comparison to the control, the co-application of biochar and N fertilizer did not affect the correlative scales of microbial colonies (Yu et al., 2018b). Nevertheless, Tan et al. (2021) indicated that straw biochar application in combination with mineral or organic fertilizer did not change the soil microbial biomass N content in this field experiment.

### 5.2 Crop production

The interaction of straw biochar and fertilizer is advocated as a good approach for improving soil qualities, thereby increasing crop biomass accumulation and crop productivity. In addition, the application of straw biochar alone may not increase crop productivity and may reduce crop yields. Therefore, straw biochar and fertilizer in combination could be a favourable management scheme for enhancing crop production in soils. The impact of straw biochar and fertilizer on crop production has been examined in a few studies (**Table S7**). The addition of straw biochar and fertilizer to soil usually promotes crop growth, such as grain yield, biomass, and root length. Incidentally, in a study, straw biochar or straw biochar combined with compost in soil administration canals promoted crop growth by improving SOC, NPK and the microbial structure of the soil, which was consistent with the outcomes of most studies. In contrast, Wang et al. (2017) found that peanut shell biochar alone or in combination with N fertilizer had little ability to increase vegetable yields, while the biomass of plants was reduced when shell biochar-based modification was combined with N fertilizer because the numerous nutrients offered inhibited root morphology. Thus, the combined application of straw biochar and fertilizer needs to be mixed in an appropriate ratio. The increases in grain yield, biomass and crop growth might have been influenced by the following factors: (a) the straw biochar application rate and soil types; (b) the improvement degree of the soil properties by straw biochar and fertilizer combination, (c) the after-effect of long-term continuous straw biochar application and (d) the retention ability of soil water and fertilizer by co-application of straw biochar and fertilizer.

## 6 Conclusions and perspectives

This article reviewed the characteristics of different straw materials and different methods of straw return and their applications. According to previous studies, the characteristics of straw are influenced by the type of straw material; therefore, they have different amounts of nutrient elements. Straw return has been utilized in a variety of patterns, such as direct straw return, straw biochar return, and their combination with fertilizer. This review discusses various impacts of straw return on soil properties and crop growth. Overall, straw return is a great method for enhancing agricultural soil quality and productivity, especially when applied with fertilizer. Straw return alters the physical, chemical, and biological properties of amended soil, thereby improving soil quality and crop production.

Future research should focus more efforts on identifying the mechanistic pathway by which soil N, P and K transformations are impacted for different soils, crops, and climates. Simultaneously, straw can be modified to meet specific crops needs. In addition, tillage methods (*e.g.*, no-tillage, conventional tillage, subsoil tillage, and rotary tillage), as agricultural management of the essential farming practices, can also strongly affect the long-term productivity of agricultural systems under complicated climatic conditions. Therefore, to gain insight into the effects of actual agricultural production, it is necessary to study the impact mechanism of combining various straw additions, tillage and fertilization on soil properties and crop growth. On the other hand, the negative behaviour of straw return towards both nutrient availability and crop response demands further insight and thus investigations to determine the most likely reasons for such an effect. Overall, the effect of straw return on crop growth mainly depends on the straw return pattern, soil, and crop. Straw return to the field is extremely important in the sustainable development of agriculture in the future. Therefore, the establishment of a continuous supply system will be needed to promote the application of straw to higher value-added areas.

## Author contributions

All of our authors discussed and proposed to revise the ranking according to their contributions. The main contributions of the authors are as follows: LC: conceptualization, investigation, formal analysis, and writing-original draft. SS: investigation and formal analysis. BY: investigation. YP: visualization. CG: investigation. TQ: investigation. YZ: conceptualization, formal analysis, and writing-original draft. CS: conceptualization, formal analysis, and writing-original draft. WQ: project administration. All authors contributed to the article and approved the submitted version.

## Funding

This study was supported by the Guangdong Province Key Field R&D Program Project (2020B0202010007), Scientific research project of Hunan Provincial Department of Education (19B275), Natural Science Foundation of Hunan Province, China (Grant No. 2018JJ3242, 2021JJ30362 and 2021JJ30361), and Science and Technology Innovation Leading Plan of High-Tech Industry in Hunan Province (Grant No. 2021GK4055).

## Conflict of interest

The authors declare that the research was conducted in the absence of any commercial or financial relationships that could be construed as a potential conflict of interest.

## Publisher’s note

All claims expressed in this article are solely those of the authors and do not necessarily represent those of their affiliated organizations, or those of the publisher, the editors and the reviewers. Any product that may be evaluated in this article, or claim that may be made by its manufacturer, is not guaranteed or endorsed by the publisher.
