# Supplementary material for: Effects of straw return and straw biochar on soil properties and crop growth: A review
Source: Front Plant Sci. 2022 Sep 27;13:986763. doi: 10.3389/fpls.2022.986763 (PMC9552067; doi:10.3389/fpls.2022.986763)
Supplement: Supplementary file 1 [file DataSheet_1.doc]

**Supplementary Information**

**Effects of straw return and straw biochar on soil properties and crop growth: A review**

Limei Chen^a^, Songlin Sun^a^, Bin Yao^b*^, Yutao Peng^c^, Chongfeng Gao^a^, Tian Qin^b^, Yaoyu Zhou^b^, Chaoran Sun^a#^, Wei Quan^a^

*^a^ School of Mechanical and Electrical Engineering, Hunan Agricultural University, Changsha, 410128, China*

*^b^ School of Resources and Environment, Hunan Agricultural University, Changsha 410128, China*

*^c^ School of Agriculture, Sun Yat-sen University, Shenzhen, Guangdong, 523758, China*

** Correspondence author at binyao121@163.com (B. Yao).*

*^#^ Correspondence author at* [*scr@hunau.edu.cn*](mailto:scr@hunau.edu.cn) *(C.R. Sun).*

This supplementary information contains 7 Tables.

**Table S1** Characteristics of various straw materials

| Crop straw | Straw yield | pH | Elemental analysis (%) | | | | | | | Reference |
| --- | --- | --- | --- | --- | --- | --- | --- | --- | --- | --- |
|  |  |  | C | N | P | K | S | Mg | Zn |  |
| maize straw | 1.2 t·ha^-1^ | - | 43.23 | 0.49 | - | - | 0.26 | 0.19 | - | (Zhang et al., 2020b) |
|  | 11.22~13.36 t·ha^-1^ (2018) | - | - | 0.87 | 0.17 | 0.9 | - | - | - | (Frank Stephano et al., 2020) |
|  | 10.02~11.58 t·ha^-1^ (2018) | - | - | 0.82 | 0.29 | 1.12 | - | - | - |  |
|  | - | - | 44.6 | 0.62 | - | - | - | - | - | (He et al., 2016) |
|  | - | - | 42.62 | 0.93 | - | - | - | - | - | (Zhao et al., 2019a) |
|  | - | - | - | 0.72 | 0.25 | 1.5 | - | - | - | (Geng et al., 2019) |
|  | - | - | 46 | 0.77 | 0.31 | 1.85 | - | - | - | (Luo et al., 2020) |
| rice straw | - | - | 42.5 | 0.75 | 0.12 | 2.42 | - | - | - | (Yuan et al., 2021) |
|  | - | - | 42.2 | 0.62 | 0.13 | 2.45 | - | - | - |  |
|  | - | - | 39.9 | 0.66 | 0.18 | 1.22 | - | - | - | (Yan et al., 2019a) |
|  | 8.42 t·ha^-1^ | - | 36.88 | 0.7 | - | - | - | - | - | (Sui et al., 2016) |
|  | - | 6.93 | 50.36 | 0.87 | 0.45 | 2.03 | - | - | - | (Cui et al., 2017) |
|  | - | 8.5 | 40.2 | 0.72 | 0.19 | 2.33 | - | - | - | (Liu et al., 2021) |
|  | - | - | - | 0.63 | 0.08 | 0.23 | - | - | - | (Zhao et al., 2016b) |
|  | - | - | 36.6 | 1.01 | - | - | - | - | - | (Shaaban et al., 2018) |
|  | - | - | - | 0.38 | 0.11 | 1.53 | - | - | - | (Bai et al., 2019) |
|  | - | 7.35 | 37.4 | 0.73 | 0.21 | - | - | - | - | (Xu et al., 2016b) |
| wheat straw | - | - | - | 0.33 | 0.08 | 1.12 | - | - | - | (Bai et al., 2019) |
|  | - | 6.8 | 37.78 | 0.76 | 0.05 | 1.65 |  |  | - | (Zhang et al., 2017) |
|  | 4.88 Mg·ha^-1^(2015) | - | 43.5 | 0.52 | 0.08 | - | - | - | - | (Zhao et al., 2019b) |
|  | 3.53 Mg·ha^-1^(2016) | - | 44.4 | 0.49 | 0.1 | - | - | - | - |  |
|  | - | - | 43.1 | 0.75 | - | - | - | - | - | (He et al., 2016) |
|  | 7.15~8.18 Mg·ha^-1^ | - | 45.6 | 0.67 | - | - | - | - | 0.57 | (Chen et al., 2021) |
|  | - | - | 48.2 | 0.55 | 0.12 | 1.15 | - | - | - | (Guo et al., 2017) |
|  | - | - | 40.4 | 1.5 | - | - | - | - | - | (Ji et al., 2016) |
|  | - | -- | 41.1 | 0.61 | 0.08 | 1.45 | - | - | - | (Yuan et al., 2021) |
|  | - | - | 41 | 0.52 | 0.09 | 1.25 | - | - | - |  |
|  | - | 7.62 | 37.9 | 0.88 | 0.26 | - | - | - | - |  |
| green bean straw | - | - | 40.1 | 2.28 | - | - | - | - | - | (Shaaban et al., 2018) |
| corn stalks | - | - | 42.3~45.6 | 0.9~0.95 | - | - | - | - | - | (Li et al., 2018a) |

Notes: “-” not measured.

**Table S2** Summary of soil response to straw return application in studies

| Straw feedstock | Crop | Soil types | Treatments | Soil depth(cm) | Impact on soil parameters | References |
| --- | --- | --- | --- | --- | --- | --- |
| maize straw | maize | eluvial brown soil | straw mixing | 0~20 | +44% (C sequestration) | (Li et al., 2019b) |
|  |  |  | straw mulching |  | +13% (C sequestration) |  |
| rice straw | rice | loam soil/clay loam soil | no-till coupled straw return(first rice reason) | 0~20 | +27.6% /+10.4% (Fe-P_i_)  +11.1% /+12.7% (Ca-P_i_)  -38.6% /-40.7% (Al-P_i_) | (Li et al., 2019a) |
| wheat straw+rice straw | rice-wheat rotation | sand loam | 50% annual straw return | 0~21 | SOC sequestration↑ | (Zhu et al., 2015a) |
| wheat straw+maize straw | wheat-maize rotation | silt clay loam | 4 t·ha^-1^ straw mulching (after three years) | 0~10 | +16.9% (SOC) /+7.7% (TN) | (Dong et al., 2018) |
|  |  |  | 4 t·ha^-1^ straw mulching (after the maize reason) |  | +24.1% (MBC) /+21.0% (DOC) /+10.5% (DON) |  |
| Maize straw | maize | loamy clay | 7.4 t·ha^-1^ straw | 0~10 | +9.30% (bulk SOC) | (Guan et al., 2019) |
| wheat straw | maize | - | straw+cattle manure+70% NPK admixture fertilizer | - | soil microbes↑ | (Ma et al., 2017) |
| wheat straw+rice straw | rice-wheat rotation | silty clay loamy | wheat +rice straw | 0~20 | >15% (AN, AP, AK) | (Zhao et al., 2019b) |
|  |  |  |  | 0~15 | +11.4% (urease activity) /+41.0% (invertase activity) /+12.9% (catalase activity) /+59% (MBC) /+54% (MBN) |  |
| wheat straw+maize straw | wheat-maize rotation | silt loam | 7.25 t·ha^-1^ wheat straw(2015~2016),  10.18 t·ha^-1^ wheat straw(2016~2017) | 0~5 | +32.0%~35.2% (SOC) | (Ma et al., 2020) |
|  |  |  |  | 5~10 | +33.1%~39.0% (SOC) |  |
|  |  |  |  | 10~20 | +53.5%~55.0% (SOC) |  |
|  |  |  | 11.43 t·ha^-1^ maize straw+9.17 t·ha^-1^ wheat straw(2015~2016), 10.97 t·ha^-1^ maize straw+10.8 t·ha^-1^ wheat straw(2015~2016) | 0~5 | +37.2%~40.8% (SOC) |  |
|  |  |  |  | 5~10 | +33.5%~45.2% (SOC) |  |
|  |  |  |  | 10~20 | +37.4%~50.7% (SOC) |  |
| maize straw+ rice straw | maize-rice rotation | - | maize straw mulched+rice straw incorporated, maize and rice straw incorporated | 0~20 | +3.6% (annual SOC) /+63.4% (DOC) /+38.8% (MBC) / +12.4% (N_min_) / +39.7% (AP) / | (Han et al., 2020) |
| corn straw | corn | sand clay loam | 8.9 t·ha^-1^ corn straw | 0~40 | +11.55%~16.58%(SOC) | (Ndzelu et al., 2021) |
| maize straw | maize-wheat rotation | sand loam | 4.5 t·ha^-1^ straw+0.36 t·ha^-1^ N+0.24 t·ha^-1^ P fertilizer | 0~20 | +11.6% (Gm^-^ bacteria abundance) | (Zhao et al., 2016a) |
|  |  |  |  |  | +68.2% (fungal abundance) |  |
|  |  |  | 9 t·ha^-1^ straw+0.36 t·ha^-1^ N+0.24 t·ha^-1^ P fertilizer |  | +9.3% (Gm^-^ bacterial abundance) |  |
|  |  |  |  |  | +113.6% (fungal abundance) |  |
| wheat straw+maize straw | maize-wheat rotation | clay soil | half straw+0.65 t·ha^-1^ N+1 t·ha^-1^ P+0.2 t·ha^-1^ K | 0~20 | -1.6 unit (pH) | (Xie et al., 2018) |
|  |  |  |  |  | +26% (SOC) |  |
| rice straw | rice-wheat rotation | - | 7.5 t·ha^-1^ straw |  | +10.2~32.5% (SOC) | (Zhao et al., 2016b) |
| wheat straw | wheat-corn rotation | sand(28%), silt(30.6%), clay(41.4%) | mineral fertilization+7.5 t·ha^-1^ straw | 0~15/15~30 | -0.3unit /-0.1 unit (pH) | (Guo et al., 2017) |
|  |  |  |  |  | +5.49% /+36.44% (SOC) |  |
|  |  |  |  |  | +10.24% /+77.51% (DOC) |  |
|  |  |  |  |  | +15.28% /+88.27% (DON) |  |
|  |  |  |  |  | +15% /+105.26% (NH_4_^+^-N) |  |
|  |  |  |  |  | +14.6% /+44.44% (NO_3_^-^-N) |  |
|  |  |  |  |  | +8.2% /+86.86% (AP) |  |
|  |  |  |  |  | +17.92% /+23.2% (AK) |  |
|  |  |  |  |  | +66.8% /+74.38% (MBC) |  |
|  |  |  |  |  | +57.3% /-28.57% (MBN) |  |
| rice straw+wheat straw | rice-wheat rotation | sand loam | 3 t·ha^-1^ straw+N fertilizer | 0~20/20~40 | -0.89/-0.33unit (pH) | (Bai et al., 2019) |
|  |  |  |  |  | +20.43/+51.36% (SOC) |  |
|  |  |  |  |  | -6.38/+31.4% (TN) |  |
| wheat straw+maize straw | wheat-maize rotation | sand soil | full straw | - | +6.07% (AK) /+23% (SOC) | (Bai et al., 2015) |
| wheat straw | green manure-winter wheat rotation | - | 9 t·ha^-1^ straw | 0~10 | +6.8% (SOC) | (Li et al., 2021a) |
|  |  |  |  | 10~20 | +4.7% (SOC) |  |
| rice straw | double-rice cropping system | sand(42.4%), silt(30.4%), clay(27.2%) | 3 t·ha^-1^ straw+NPK fertilizer | 0~20 | +7.36% (SOC) /+2.47% (TN) /+3.57% (TP) /+0.13unit (pH) | (Liu et al., 2021) |
|  |  |  | 6 t·ha^-1^ straw+NPK fertilizer |  | +9.4% (SOC) /+4.94% (TN) /+1.79% (TP) /+0.16unit (pH) |  |
| wheat straw | rice-wheat rotation | clay | 3 t·ha^-1^ straw(controlled irrigation) | 0~40 | +23.2%(SOC) | (Yang et al., 2018) |
| rice straw+ wheat straw | rice-wheat rotation | loamy | 6 t·ha^-1^ wheat straw+8 t·ha^-1^ rice straw | 0~20 | +13.8~22.3%(SOC) | (Chen et al., 2018) |
|  |  |  | 5 t·ha^-1^ wheat straw+8.5 t·ha^-1^ rice straw | 0~10 | +32%(SOC) |  |
| rice straw+ wheat straw | rice-wheat rotation | loam | 7.81 t·ha^-1^ rice straw+5.7 t·ha^-1^ wheat straw | 0~20 | +7.95~25%(SOC) | (Yuan et al., 2021) |
|  |  | silty loam | 8.01 t·ha^-1^ rice straw+4.73 t·ha^-1^ wheat straw |  |  |  |
| wheat straw+ corn straw | wheat-maize rotation | - | 14.301 t·ha^-1^ straw | 0~20 | -0.1 unit (pH) /+25.55% (SOC) /+89.29% (TN) /+75.78% (AP) /+3.35% (AK) | (Chen et al., 2017) |
|  |  |  | 14.301 t·ha^-1^ straw+0.28 t·ha^-1^ N fertilizer |  | +120.52% (SOC) /+53.57% (TN) /+291% (AP) /+285.8% (AK) |  |
| Wheat straw |  |  | 14.301 t·ha^-1^ straw+0.28 t·ha^-1^ N fertilizer |  | +74.85% (SOC) /+126.79% (TN) /+194.04% (AP) /+229.1% (AK) |  |
| corn stalks | wheat | clay loam | NPK fertilizer+pig manure(7.5 t·ha^-1^)+ straw return | 0~20 | TN, SOC↑(from 2008 onwards) | (Li et al., 2018a) |
| rice straw | rice | silty clay loam | 7.1~10.6 t·ha^-1^ straw+2.9~4.2 t·ha^-1^ green manure+0.04 t·ha^-1^ P fertilizer | 0~20 | +13~20% (P accumulation) | (Li et al., 2015) |
| rice straw | rice-rice-green manure rotation | silt loam | full straw | 0~20 | *nifH* gene abundance↓ | (Yang et al., 2019) |
|  |  |  |  |  | diazotrophic bacteria species↑ |  |
| maize straw | wheat-maize | silty clay loam | 0.45 t·ha^-1^ N+0.15 t·ha^-1^ P+10.5 t·ha^-1^·yr^-1^straw | 0~10 | +53% (mineralization C) | (Li et al., 2018c) |
| rice straw | maize and ryegrass | sand (7.0%), silt (71.6%), clay (22.4%) | 15.6 t·ha^-1^ | - | SOC↑, AN↓ | (Xu et al., 2016b) |
| wheat straw |  |  |  |  | SOC, AN↓ |  |
| wheat straw+maize straw | wheat-maize rotation | - | all straw+285 kg·ha^-1^ N (wheat season) | 0~20 | Average + 12.9% (SOC) | (Yang et al., 2015) |
|  |  |  | all straw+210 kg·ha^-1^ N (maize season) |  | Average + 6.1% (TN) |  |

Notes: all treatments are compared with control (without straw return). “↑” means increased, “↓” means decreased. “-” not measured. Soil organic carbon (SOC), total nitrogen (TN), phosphorus (TP), total potassium (TK), microbial biomass carbon (MBC), microbial biomass nitrogen (MBN), dissolved organic carbon (DOC), dissolved organic nitrogen (DON), available nitrogen (AN), available phosphorus (AP), available potassium (AK), mineralized nitrogen (N_min_), dissolved organic nitrogen(DON). The same blow.

**Table S3** Summary of crop responses to straw return application in studies

| Straw feedstock | Test crop | Soil types | Treatments | Crop responses | References |
| --- | --- | --- | --- | --- | --- |
| wheat straw | wheat-maize rotations | silty clay loam | 8 t·ha^-1^ wheat straw +fertilizer | +63.1%/ +49.8% (wheat/maize yield) | (Hu et al., 2021) |
| wheat straw+rice straw | rice-wheat rotation | sand loam | 50% annual straw return | +13.0%~17.12% (crop yield) | (Zhu et al., 2015a) |
| wheat straw+maize straw | wheat-maize rotation | silt clay loam | 4 t·ha^-1^ straw mulching | +9.8% (average maize yield) | (Dong et al., 2018) |
|  |  |  |  | +7.4% (average wheat yield) |  |
| maize straw | maize | Alifisol | 4 t·ha^-1^ straw amendment | +11.0% (average yield) | (Jiang and Yu, 2019) |
|  |  |  | 8 t·ha^-1^ straw amendment | +12.8% (average yield) |  |
| maize straw | maize | chernozem | 1.2 t·ha^-1^ straw+15 kg·ha^-1^ magnesium fertilizer | +5.28% (yield) | (Zhang et al., 2020b) |
| wheat straw+maize straw | wheat-maize rotation | - | wheat +maize straw | +32.5% (average maize yield) | (Li et al., 2019c) |
|  |  |  |  | +50.5% (average wheat yield) |  |
| wheat straw+rice straw | rice-wheat rotation | silty clay loamy | wheat +rice straw | +58% ( average wheat yield) | (Zhao et al., 2019b) |
| maizes traw+ rice straw | maize-rice rotation | - | maize straw mulched+rice straw incorporated | +9.1%/+11.7% (annual yield in 2017/2018) | (Han et al., 2020) |
|  |  |  | maize and rice straw incorporated | +15.2%/+12.9% (annual yield in 2017/2018) |  |
| maize straw | maize | Chernozem soil | 12 t·ha^-1^ straw +Silicon | +6.3%(yield) | (Frank Stephano et al., 2020) |
| maize straw | maize | - | straw+0.9 t·ha^-1^ K fertilizer | +1.35~71.01%(root length)/ +19.16~42.45%(root surface area) /+10.49~22.73%(root volume) /+4.43~7.05%(yield) | (Ya et al., 2021) |
| wheat straw | maize/wheat | desert soil | straw covering | +27~38% /+153~160% (yield) | (Yin et al., 2018) |
| maize cobs | wheat-maize rotation | - | 15Mg·ha^-1^ straw | +1.18% (maize yield) | (He et al., 2016) |
| maize straw+ rice straw | maize-rice rotation | paddy soil | straw+33% K fertilizer | +1.5% (annual yield) | (Han et al., 2021) |
|  |  |  | straw+67% K fertilizer | +3.2% (annual yield) |  |
| maize straw | maize | sand loam | straw+N fertilizer (2015~2016) | +42.84~63.36 (yield) | (Xu et al., 2018) |
|  |  |  | straw+N fertilizer (2016~2017) | +49.05~63.06 (yield) |  |
| wheat straw+maize straw | maize/Welsh onion-wheat rotation | silt loam | straw return | +7~16% (yield) | (Yao et al., 2017) |
| soybean straw+wheat straw | wheat-soybean system | silty clay loam | 0.7 t·ha^-1^ soybean straw+0.3 t·ha^-1^ wheat straw | +6.9~7.8% (wheat yield) | (Wang et al., 2019) |
|  |  |  |  | +13.3% (soybean yield in 2014), +21.6% (soybean yield in 2015) |  |
|  |  |  | 1.4 t·ha^-1^ soybean straw+0.6 t·ha^-1^ wheat straw | +13.4~13.9% (wheat yield) |  |
|  |  |  |  | +19.2~39.6% (soybean yield) |  |
| maize stalk | wheat-maize rotation | loam soil | half straw return | +17.1% (maize yield) /+6.3% (kernels) /+11% (TKW) | (Gao et al., 2019) |
|  |  |  | all straw return | +15.1% (maize yield) /+8.5% (kernels) /+6% (TKW) |  |
| maize straw | maize | silty loam | 3.23, 6.45, 9.68 and 12.9 t·ha^-1^ straw | +44.59, 45.65, 48.60 and 31.45% (yield in 2015) | (Geng et al., 2019) |
|  |  |  |  | +50.15, 52.47, 52.22 and 40.86% (yield in 2016) |  |
| maize straw | maize | chernozem | 1.2 t·ha^-1^ straw+0.015 t·ha^-1^ magnesium fertilizer | +5.3% (yield) | (Zhang et al., 2020a) |
| wheat straw+maize straw | wheat-maize rotation | sand soil | full straw | +16.5% / +13.2% (average wheat yield / maize yield) | (Bai et al., 2015) |
|  |  |  |  | +15.9% /+21.8% (the K content in wheat straw/maize straw) |  |
|  |  |  |  | +32.7% / +30.9% (TK uptake by wheat / maize) |  |
| maize straw | lettuce-cabbage-chili-lettuce | clay loam | 50%straw+mineral fertilizer | +55.95%(total yield) | (Huang et al., 2019) |
|  |  |  | 60%straw+mineral fertilizer | +67.38%(total yield) |  |
|  |  |  | 70%straw+mineral fertilizer | +64.1%(total yield) |  |
|  |  |  | 100%straw+mineral fertilizer | +75.6%(total yield) |  |
| rice straw | rice | clay | 2.625 t·ha^-1^ straw | +14.5~23.6%(yield in 1981 to 2019) | (Song et al., 2021) |
| maize straw | maize | - | 10 t·ha^-1^ straw | +2.48~24.46%(yield) | (Tian et al., 2020) |
| wheat straw | maize | silty loam | 4 t·ha^-1^ straw | +4.8% (average TKW) /+3.4% (average yield) | (Li et al., 2021b) |
|  |  |  | 4 t·ha^-1^ ammoniated straw | +8.26% (average TKW) /+8.2% (average yield) /+2.55% (average aboveground biomass) |  |
| wheat straw | wheat | silty clay loam | 0.105 kg/pot | -37.3% (yield) | (Ji et al., 2016) |
|  |  |  | 0.35 kg/pot | -90.1% (yield) |  |
| rice straw | rice | - | 6 t·ha^-1^ straw | +1.2% (yield) | (Cui et al., 2017) |
| rice straw+ wheat straw | rice-wheat rotation | loamy | 6 t·ha^-1^ wheat straw | +11.6% (rice yield) | (Chen et al., 2018) |
|  |  |  | 8 t·ha^-1^ rice straw | +11.1% (wheat yield) |  |
| rice straw+ wheat straw | rice-wheat rotation | loam | 7.81 t·ha^-1^ rice straw+5.7 t·ha^-1^ wheat straw | +12.52% (rice yield)/+20.64% (wheat yield) | (Yuan et al., 2021) |
|  |  | silty loam | 8.01 t·ha^-1^ rice straw+4.73 t·ha^-1^ wheat straw | +11.81% (rice yield)/+5.97% (wheat yield) |  |
| wheat straw+ corn straw | wheat-maize rotation | - | 14.301 t·ha^-1^ straw | +8% (maize yield) | (Chen et al., 2017) |
|  |  |  | 14.301 t·ha^-1^ straw+0.28 t·ha^-1^ N fertilizer | +154% and 116% (maize yield) |  |
| wheat straw+ corn straw/maize straw | maize | - | 7.571 t·ha^-1^ straw+53 kg·ha^-1^ N+12.1 kg·ha^-1^ P_2_O_5_+56.8 kg·ha^-1^ K_2_O | +179.55% and 273.58% in 2016 and 2017 (yield) | (Lv et al., 2019) |
|  |  |  |  | +149.54% and 137.56% in 2016 and 2017 (kernel) |  |
|  |  |  |  | +26.96% and 41.42% in 2016 and 2017 (TKW) |  |
| corn stalks | wheat | silty loam | NPK fertilizer+straw return | +13.2% (yield) | (Li et al., 2018a) |
|  |  |  | NPK fertilizer+pig manure(7.5 t·ha^-1^)+ straw return | +17.4%(yield) |  |
| maize straw | maize | - | maize straw+0.25 t·ha^-1^ N fertilizer(2017/2018) | +336.93/364.41% (yield) /+282.86/398.39% (aboveground biomass) /+447.8/689.19% (aboveground N uptake) | (Bai et al., 2021) |
| wheat straw | maize | silt loam | 3 t·ha^-1^ compost+70% NPK+6Mg·ha^-1^ (average) | 7~15% (yield) | (Zhang et al., 2016b) |
| rice straw | rice | - | 6 t·ha^-1^ straw +0.15 t·ha^-1^ N fertilizer | 68.7~115.9% (yield) | (Chen et al., 2020) |

Notes: thousand kernel weight (TKW).

**Table S4** Summary of soil response to straw biochar return application in studies

| Biochar feedstock | Pyrolysis temp(℃) | Elemental analysis (%) | | | | pH | Crop | Soil types | Treatment | Impact on soil parameters | References |
| --- | --- | --- | --- | --- | --- | --- | --- | --- | --- | --- | --- |
|  |  | C | N | P | K |  |  |  |  |  |  |
| maize straw | 450 | 50.69 | 0.85 | - | 0.97 | 9.41 | maize | loamy clay | 6.3 t·ha^-1^ | +23.4% (bulk SOC) | (Guan et al., 2019) |
|  | 360 | 65.7 | 0.91 | - | - | 8.2 | wheat-maize rotation | Sand (73.5%),silt (12.5%), clay (14%) | 4.5 and 9 t·ha^-1^ | +61.0~116.3% (SOC) | (Zhou et al., 2018) |
| corn straw | 450 | 71.5 | 1.54 | 0.78 | 1.68 | 7.94 | soybean | - | 2.5, 5, 10% | SOC, TN, AP, AK↑ | (Liu et al., 2020a) |
| wheat straw | 650 | 51.1 | 1.7 | - | 0.16 | - | wheat | silty clay loam | 0.028, 0.084, 0.28 kg/pot | +13.2~155.1% (average SOC); MBC↑, DOC↓ (0.028, 0.084 kg/pot) | (Ji et al., 2016) |
| maize straw | 400 | 59.16 | 0.98 | - | - | 9.8 | maize | clay loam | 10, 20, 30 t·ha^-1^ | SOC, TN, AP, AK↑ | (Xiao et al., 2016) |
| rice straw | 400~500 | 63.27 | 1.28 | 0.73 | 1.46 | 9.02 | rice | - | 2 t·ha^-1^ | NH_4_^+^-N↑/+62.9% (SCS) | (Cui et al., 2017) |
|  |  |  |  |  |  |  |  |  | 40 t·ha^-1^ | NO_3_^-^-N↑/+214% (SCS) |  |
| maize stalk | 400 | 68.5 | 1.34 | 0.15 | 1.34 | 9.6 | - | loamy sand | 3.2, 16, 32 t·ha^-1^ | +31~298% (SOC) | (Lin et al., 2015) |
| maize straw | 500 | 62.45 | 1.1 | - | - | 10.42 | maize | sand (34%), silt (26%), clay (40%) | 0.6 t·ha^-1^ | +0.39 units (pH)/+27.8% (SOC)/+15.31%(TN)/+3.26% (TP)/+8.24%(TK)/+8.7% (NO_3_^-^-N)/+2.33%(NH_4_^+^-N)/+9.64%(AP)/+26.09%(AK) | (Qiao et al., 2020) |
| wheat straw | 350~550 | 46.72 | 0.59 | 1.44 | 1.15 | 9.9 | tomato | loam | 25, 50 t·ha^-1^ | SOM, TN↑ | (Agbna et al., 2017) |
|  |  |  |  |  |  |  |  |  |  | NH_4_^+^-N, NO_3_^-^-N↓ |  |
| rice straw | 450 | 67.07 | 0.81 | - | - | 9.1 | rice | - | 14.8/29.6 t·ha^-1^ | +0.58%/ +1.17% (SOC) | (Sui et al., 2016) |
| cotton straw | 800 | 68.7 | 0.325 | 0.12 | 1.6 | 8.6 | wheat-maize rotation | - | 10 t·ha^-1^ | +16.8% in the wheat season (abundances of *Nitrosovibrio*) | (Shi et al., 2019b) |
| rice straw | 400~450 | 88.82 | 0.25 | 0.08 | 1.17 | 9.6 | tomato | sand (47.57%), silt (35.06%), clay (17.36%) | 0.37 kg·ha^-1^ straw+9.5 t·ha^-1^ biochar | pH↑/+40.3% (AN)/+157.2% (AP)/+24.2% (AK) | (Zhang et al., 2018) |
| corn straw | 400~450 | 50.6 | 1.4 | 0.46 | 1.78 | 8.56 | soybean | Planosol | 10/20/30 g/pot (compared to straw return) | +6.7% (pH)/SOM, AN, AP, AK↑/+9.83% (TN)/+25.56% (TP) | (Xiu et al., 2019) |
| maize straw | 450 | 53.81 | 1.22 | - | 1.67 | 10.5 | - | - | 0.5, 1.0, 2.5, 5.0% by mass | total phospholipid fatty acid content, the relative abundance of bacteria↑ | (Wang et al., 2015a) |
| maize straw | 300 | 48.9 | 1.25 | - | - | 9.84 | - | fluvo-aquic soil | 1% biochar (by soil mass) | SOC, TN, NH_4_^+^-N↑  NO_3_^-^-N first ↑ and then ↓with increasing pyrolysis temperature | (Wang et al., 2015b) |
|  | 450 | 53.81 | 1.22 | - | - | 10.47 |  |  |  |  |  |
|  | 600 | 62.9 | 1.28 | - | - | 11.37 |  |  |  |  |  |
| maize straw | 400 | 76 | 5 | - | 4 | 10.7 | - | sand (52%), silt (18%), clay (30%) | 12 g/pot | +43 mg·kg^-1^ (AP)/+8 mg·kg^-1^ (SMB-P) | (Zhai et al., 2015) |
|  |  |  |  |  |  |  |  | sand (36%), silt (29%), clay (35%) |  | +124 mg·kg^-1^ (AP)/+12 mg·kg^-1^ (SMB-P) |  |
| maize straw | 300 | 48.9 | 1.25 | 0.06 | 3.34 | 9.84 | wheat | - | 40.5g/pot | +32.09~35.73% (TN), +188.34~260.06% (SOC),+19.57~20.09% (AP),+280.04~343.31% (AK), | (Song et al., 2018) |
|  | 450 | 53.81 | 1.22 | 0.06 | 6.61 | 10.47 |  |  |  |  |  |
|  | 600 | 62.9 | 1.21 | 0.06 | 5.49 | 11.37 |  |  |  |  |  |
| maize straw | 400~500 | 67.57 | - | - | - | 9.0 | tomato | Sand (64.1%), silt (16.5%), clay (19.4%) | 10/20/40/60 t·ha^-1^ | +0.2 unit (pH)/AN, AP, AK↑ | (Li et al., 2018b) |
|  |  |  |  |  |  |  |  |  | 60 t·ha^-1^ | two-fold↑ (SOM) |  |
| wheat straw | 400 | 48.65 | 0.75 | 0.17 | 4.92 | 9.2 | wheat-maize rotation | Sand (46.3%), silt (31.1%), clay (22.6%) | 2.25/6.75/11.25 Mg·ha^-1^ | SOC, TN, NO_3_^-^, AK, *amoA* gene abundance↑ | (Xie et al., 2021) |
| rice straw | 400 | 43.5/49.4 | 1.1/1.31 | 0.21 | 5.96/6.32 | 9.98/9.85 | maize | red soil | 24 t ha^-1^ | +0.13 units (pH), +8.16/+6.12% (N content) | (Zhu et al., 2015b) |
| corn stover | 450~480 | 62.1 | 0.303 | 0.131 | 0.182 | 10.2 | tobacco | - | 1/5/10/20 Mg·ha^-1^ | pH, SOC, LOC↑ | (Zhang et al., 2019) |
|  |  |  |  |  |  |  |  |  |  | abundance of actinomyces↑ |  |
| peanut shell | 500 | 64.7 | 1.522 | - | - | 9.16 | wheat-maize rotation | yellow cinnamon  soil | 4.5 t·ha^-1^ | +53.15% (MBC)/+17.72% (AP)/+12.24% (AK)/+15.73% (SOC) | (Liu et al., 2020b) |

Notes: “-” not measured. soil carbon sequestration (SCS), soil organic matter (SOM), soil microbial biomass P (SMB-P), labile organic carbon (LOC).

**Table S5** Summary of crop response to straw biochar return application in studies

| Biochar feedstock | Pyrolysis temp(℃) | Elemental analysis (%) | | | | pH | Crop | Soil types | Treatment | Crop responses | References |
| --- | --- | --- | --- | --- | --- | --- | --- | --- | --- | --- | --- |
|  |  | C | N | P | K |  |  |  |  |  |  |
| maize cobs | 360 | 65.7 | 0.91 | 0.08 | 1.6 | - | wheat-maize rotation | Fluvic Cambisol | 9 t·ha^-1^ | +4.18% (wheat yield) | (He et al., 2016) |
| corn straw | 450 | 71.5 | 1.54 | 0.78 | 1.68 | 7.94 | soybean | - | 2.5%/5%/10% (w/w) | +1.5 cm/+4.8 cm/+3.6 cm (height) | (Liu et al., 2020a) |
|  |  |  |  |  |  |  |  |  |  | +1.71%/+4.49%/+4.31% (100-seed weight) |  |
|  |  |  |  |  |  |  |  |  |  | +5.47%/+11%/+7.57% (seed yield) |  |
|  |  |  |  |  |  |  |  |  |  | +2.49%/+8.09%/+7.4% (biomass) |  |
| wheat straw | 650 | 51.1 | 1.7 | - | 0.16 | - | wheat | silty clay loam | 0.028/0.084 kg/pot | +14.9%/+19.1% (yield) | (Ji et al., 2016) |
| rice straw | 400~500 | 63.27 | 1.28 | 0.73 | 1.46 | 9.02 | rice | - | 2/40 t·ha^-1^ | +2.9%/+5.2% (yield) | (Cui et al., 2017) |
| maize stalk | 400 | 68.5 | 1.34 | 0.15 | 1.34 | 9.6 | soya bean/wheat | loamy sand | 16 t·ha^-1^ | +11/+32% (aboveground biomass per plant) | (Lin et al., 2015) |
|  |  |  |  |  |  |  |  |  |  | +24/+28% (grain mass per plant) |  |
| maize straw | 550 | 57.17 | 0.61 | 0.12 | 1.3 | 8.9 | wheat | silty loam | 20, 40, 60 t·ha^-1^ | +19.5/+25.5/+27% (aboveground biomass) | (Yan et al., 2019b) |
|  |  |  |  |  |  |  |  |  |  | +6/+13.5/+6.7% (yield) |  |
| corn straw | 500~600 | 70.08 | 1.68 | 0.82 | 1.55 | - | maize | - | 30 t·ha^-1^ | +16.5% (average photosynthetic rate) | (Feng et al., 2021) |
|  |  |  |  |  |  |  |  |  |  | +100% (average transpiration rate) |  |
|  |  |  |  |  |  |  |  |  |  | +21% (average chlorophyll value) |  |
|  |  |  |  |  |  |  |  |  | 20 t·ha^-1^ | +11.9% (yield) |  |
| wheat straw | 350~550 | 46.72 | 0.59 | 1.44 | 1.15 | 9.9 | tomato | loam | 25, 50 t·ha^-1^ | yield↑ | (Agbna et al., 2017) |
| corn straw | 400~450 | 50.6 | 1.4 | 0.46 | 1.78 | 8.56 | soybean | Planosol | 10/20/30 g/pot | +10% (yield) | (Xiu et al., 2019) |
| corn stover | 450~480 | 62.1 | 0.303 | 0.131 | 0.182 | 10.2 | tobacco | - | 1/5/10/20 Mg·ha^-1^ | total sugar, sugar contents↓ | (Zhang et al., 2019) |
|  |  |  |  |  |  |  |  |  |  | leaf K content, leaf quality↑ |  |
| peanut shell | 500 | 64.7 | 1.522 | - | - | 9.16 | wheat-maize rotation | yellow cinnamon  soil | 4.5 t·ha^-1^ | +11.52% (seed number) | (Liu et al., 2020b) |
|  |  |  |  |  |  |  |  |  |  | -1.38% (thousand seed weight) |  |
|  |  |  |  |  |  |  |  |  |  | +7.25% (seed number) |  |
| peanut shell | 550±50 | 67.4 | 1.3 | - | - | 10.1 | peanut | - | 85 t·ha^-1^ | +2-and 3-folds (peanut biomass and pod yield) | (Xu et al., 2015) |

**Table S6** Summary of soil response to fertilizer and straw biochar return application in studies

| Biochar feedstock | Pyrolysis temp(℃) | Elemental analysis (%) | | | | pH | Crop | Soil types | Treatment | Soil depth(cm) | Impact on soil parameters | References |
| --- | --- | --- | --- | --- | --- | --- | --- | --- | --- | --- | --- | --- |
|  |  | C | N | P | K |  |  |  |  |  |  |  |
| rice/wheat straw | 500~600 | - | 1.71/0.84 | 0.28/0.23 | 3.92/4.44 | - | rice-wheat rotation | sand loam | 1 t·ha^-1^r+N fertilizer | 0~20/  20~40 | -0.30/-0.08unit (pH)/+63.91/+67.37% (SOC)/-8.25/+16.28% (TN) | (Bai et al., 2019) |
| wheat straw | - | 41.8 | 0.58 | 0.12 | 0.92 | 9.3 | double-rice cropping system | Sand (42.4%), silt (30.4%), clay (27.2%) | 24 t·ha^-1^ +NPK fertilizer | 0~20 | +31.48% (SOC)/+6.17% (TN)/+8.93% (TP)/+0.11unit (pH) | (Liu et al., 2021) |
|  |  |  |  |  |  |  |  |  | 48 t·ha^-1^ +NPK fertilizer |  | +53.02% (SOC)/+9.26% (TN)/+12.5% (TP)/+0.44unit (pH) |  |
| maize straw | 300 | 48.9 | 1.25 | 0.06 | 3.34 | 9.84 | wheat | - | NPK fertilizer+50 g/pot | - | -0.02~0.14 units (pH), +198.35~272.64% (SOC), +38.98~44.07% (TN), +7.17~18.7% (DOC), +96.54~112.24% (AP), +436.79~526.17% (AK) | (Song et al., 2018) |
|  | 450 | 53.81 | 1.22 | 0.06 | 6.61 | 10.47 |  |  |  |  |  |  |
|  | 600 | 62.9 | 1.21 | 0.06 | 5.49 | 11.37 |  |  |  |  |  |  |
| wheat straw | 400 | 46.2 | 0.72 | 0.12 | - | 9.2 | rice-wheat rotation | silty clay loam | 0.25 t·ha^-1^ N fertilizer+20/40 t·ha^-1^ | 0~20 | +26~53% (SOC)/+14~16% (TN)/+6~19% (TP)/-13.2~74.7% (NO_3_^-^-N) | (Zhang et al., 2020c) |
| wheat straw | 450 | 45.75 | 0.89 | - | - | 9.92 | soybean | Ultisols | 19.5 t·ha^-1^ r+30 kg·ha^-1^ N fertilizer | 0~15 | total microbial biomass, TN↑ | (Yu et al., 2018) |
|  |  |  |  |  |  |  |  |  |  |  | +0.6~1.2 units (pH)/+38.5~54.7 mg·ka^-1^ (IN)/+136~452 mg·ka^-1^ (TN)/23.6~40.9 nmol·g^-1^(PLFAs) | (Yu et al., 2016) |
| maize straw | 500 | - | - | - | - | - | - | - | 5 g·kg^-1^ +0.5 mg·kg^-1^ oxalic acid | - | urease, polyphenol oxidase and dehydrogenase were the highest | (Li et al., 2019e) |
|  |  |  |  |  |  |  |  |  |  |  | soil microbial biomass and the abundances of genera↑ |  |
| cottonseed husk | 450 | 35.9 | 0.45 | - | - | 9.7 | peanut | - | 50 t ha^-1^ +mineral fertilizer or +0.9 t ha^-1^ organic fertilizer | 0~20 | +0.51~0.6 units (pH)  +83.02~116.32% (SOM)  +67.69~126.15% (NH_4_^+^) | (Tan et al., 2021) |
| rice husk |  | 49.6 | 0.44 | - | - | 10.3 |  |  |  |  |  |  |
| rice straw | 600 | 56.6 | 1.4 | 1.0 | 1.8 | - | early rice | silt | 8 t ha^-1^ r+8 t ha^-1^ steel slag | 0~30 | +28.7% (SOC) | (Wang et al., 2018) |
|  |  |  |  |  |  |  | late rice |  |  |  | +42.2% (SOC) |  |
| corn straw | 500 | 58 | 2.3 | - | - | 10 | - | - | 2, 4, 8% (mass ratio)+250 kg·ha^-1^ | - | -18.8~20.2% (N leaching) | (Xu et al., 2016a) |
|  |  |  |  |  |  |  |  |  |  |  | microbial biomass, pH, bacterial diversity↑ |  |
|  |  |  |  |  |  |  |  |  |  |  | the relative abundance of *Acidobacteria*, *Chloroflexi*,*Gemmatimonadetes*↓; *Proteobacteria*, *Bacteroidetes*, *Actinobacteria*↑ |  |
| wheat straw | 450 | 46.7 | 0.59 | 1.44 | 1.15 | 10.4 | Sand (29.3%),silt (39.1%),clay (31.6%) | rapeseed-sweet potato rotation | 20, 40 t ha^-1^+N fertilizer (60, 90, 120 kg·ha^-1^) | 0~15 | pH, AP↑ | (Jin et al., 2019) |
| cotton straw | 800 | 68.7 | 0.325 | - | - | 8.6 | aquic incept sol | wheat | 120 g/pot+120 g/pot organic fertilizer | - | *nirS* and *nirK* genes copies↓, the *nosZ* gene copies↑ | (Shi et al., 2019a) |
| wheat straw | 350~550 | 73.7 | 1.013 | 0.158 | 0.823 | 9.31 | sand loam | wheat | 2% +NPK | - | NH_4_^+^-N, TN, AP, AK↑ | (Liu et al., 2019) |
|  |  |  |  |  |  |  |  |  |  |  | α-diversity of bacteria↑ |  |
|  |  |  |  |  |  |  |  |  |  |  | relative abundances of Proteobacteria, Actinobacteria, and Bacteroidetes↑ |  |
|  |  |  |  |  |  |  |  |  |  |  | -61% (relative abundance of Firmicutes) |  |
| peanut shell | 500 | 64.7 | 1.522 | - | - | 9.16 | yellow cinnamon  soil | wheat-maize rotation | 4.5 t·ha^-1^ +9 t·ha^-1^ chicken manure | 0~20 | +198.19% (MBC)/+0.02 units (pH)/-12.27% (AN)/+174% (AP)/+13.92% (AK)/+21.76% (SOC) | (Liu et al., 2020b) |
| rice straw | 600 | 53.7 | 1.2 | - | - | 10.2 | sand loam | - | 1g /pot+N fertilizer | - | the abundance of ammonia-oxidizing bacteria, the relative abundance  of 60 bp T-RF (*Nitrosospira* cluster 3a and cluster 0) ↑ | (Bi et al., 2017) |
|  |  |  |  |  |  |  |  |  |  |  | the relative abundance  of 156 bp T-RF (*Nitrosospira* cluster 3c)↓ |  |
| rice straw | 550~650 | 44.268 | 0.163 | - | - | 9.71 | silt loam | rice | 2.25 t·ha^-1^ +NP fertilizer | 0~15 | pH, TN↑ | (Si et al., 2018) |

Notes: total inorganic N (IN), microbial phospholipid fatty acids (PLFAs).

**Table S7** Summary of crop responses to fertilizer and straw biochar return application in studies

| Biochar feedstock | Pyrolysis temp(℃). | Elemental analysis (%) | | | | pH | Soil types | Test crop | Treatments | Crop responses | References |
| --- | --- | --- | --- | --- | --- | --- | --- | --- | --- | --- | --- |
|  |  | C | N | P | K |  |  |  |  |  |  |
| wheat straw | 350~550 | 48.96 | 1.07 | 0.20 | 2.63 | 10.3 | silty clay loam | wheat-maize rotations | 8 t·ha^-1^ +fertilizer | +66.3 (wheat yield)  +65.4 (maize yield), CH↑ | (Hu et al., 2021) |
|  |  |  |  |  |  |  |  |  | 16 t·ha^-1^ +fertilizer | +81.7 (wheat yield)  +72.1 (maize yield), CH↑ |  |
| maize straw | 300 | 48.9 | 1.25 | 0.06 | 3.34 | 9.84 | - | wheat | NPK fertilizer+50 g/pot | +10.6~24.2% (wheat yield), +14.7~24.1% (N uptake), +12~24.8% (P uptake), +20.3~33.7% (K uptake) | (Song et al., 2018) |
|  | 450 | 53.81 | 1.22 | 0.06 | 6.61 | 10.47 |  |  |  |  |  |
|  | 600 | 62.9 | 1.21 | 0.06 | 5.49 | 11.37 |  |  |  |  |  |
| wheat straw | 400 | 46.2 | 0.72 | 0.12 | - | 9.2 | silty clay loam | rice-wheat rotation | 0.25 t·ha^-1^ N fertilizer+20/40 t·ha^-1^ (compared with N fertilization) | +3~19% (rice and wheat root length)/+10~16% (grain biomass)/+20~53% (grain NUE)/+38~230% (grain PUE)/-2~5% (N stocks in the root) | (Zhang et al., 2020c) |
| rice straw | 550 | 51.3 | 0.63 | 0.12 | 2.82 | 10.1 | clay loam | rice | 0.12 t·ha^-1^ N fertilizer+30% urea+2.8 t·ha^-1^ | yield, total biomass accumulation, greenness of the leaves↑ | (Yang et al., 2020) |
| - | 450 | 67.02 | 0.57 | 0.18 | 0.6 | 9.67 | silty clay | wheat | 20 t·ha^-1^ +N fertilizer | +12.2~13.8% (wheat biomass) | (Li et al., 2019d) |
| cottonseed husk | 450 | 35.9 | 0.45 | - | - | 9.7 | - | peanut | 50 t ha^-1^ cottonseed husk +mineral fertilizer (0.89 t ha^-1^ CO (NH_3_)_2_, 0.34 t ha^-1^ P_2_O_5_, and 0.23 t ha^-1^ K_2_O) or +0.9 t ha^-1^ organic fertilizer | +51.46~58.48% (yield) | (Tan et al., 2021) |
| rice husk |  | 49.6 | 0.44 | - | - | 10.3 |  |  | 50 t ha^-1^ rice husk +mineral fertilizer (0.89 t ha^-1^ CO (NH_2_)_2_, 0.34 t ha^-1^ P_2_O_5_, and 0.23 t ha^-1^ K_2_O) or +0.9 t ha^-1^ organic fertilizer |  |  |
| rice straw | 400 | 43.5/49.4 | 1.1/1.31 | 0.21 | 5.96/6.32 | 9.98/9.85 | red soil | maize | 24 t ha^-1^ +NPK (0.15, 0.1, 0.15 g·kg^-1^) | maize biomass, NUE↑ | (Zhu et al., 2015b) |
| wheat straw | 450 | 46.7 | 0.59 | 0.008 | 2.6 | 10.4 | calcareous incept sol | maize | 20/40 t ha^-1^ +217 kg·ha^-1^ N+145.5 kg·ha^-1^ P_2_O_5_ | yield, partial nutrient productivity↑ | (Zhang et al., 2016a) |
| wheat straw | 450 | 46.7 | 0.59 | 1.44 | 1.15 | 10.4 | Sand (29.3%), silt (39.1%), clay (31.6%) | rapeseed-sweet potato rotation | 20, 40 t ha^-1^+N fertilizer (60, 90, 120 kg·ha^-1^) | yield↑ | (Jin et al., 2019) |
| wheat straw | 350~550 | 73.7 | 1.013 | 0.158 | 0.823 | 9.31 | sand loam | wheat | 2% +N [0.943g·kg^-1^ (NO4)_2_SO_4_]P (0.42 g·kg^-1^ KH_2_PO_4_)K(0.21 g·kg^-1^ K_2_SO_4_) | grain yield and total  biomass↑ | (Liu et al., 2019) |
| rice straw | 400~500 | 28 | 1.7 | 0.28 | - | 10 | sand(49.4%),silt(20.1%),clay(30.5%) | canola | 3.75 t ha^-1^ +150 kg·ha^-1^ urea+120 kg·ha^-1^ P_2_O_5_+120 kg·ha^-1^ K_2_O | canola growth, seed, straw yield↑ | (Zhao et al., 2020) |
| canola straw |  | 49 | 2.3 | 0.55 | - | 9.49 |  |  |  |  |  |
| peanut straw |  | 44 | 2.9 | 0.21 | - | 10.54 |  |  |  |  |  |
| peanut shell | 500 | 64.7 | 1.522 | - | - | 9.16 | yellow cinnamon  soil | wheat-maize rotation | 4.5 t·ha^-1^ +9 t·ha^-1^ chicken manure | +13.57% (seed number)/-3.8% (thousand seed weight)/+12.76% (yield) | (Liu et al., 2020b) |
| rice straw | 550~650 | 44.268 | 0.163 | - | - | 9.71 | silt loam | rice | 2.25 t·ha^-1^ +N (180 kg·ha^-1^ N) P (67.5 kg·ha^-1^ P_2_O_5_) K (67.5 kg·ha^-1^ K_2_O) | yield↑ | (Si et al., 2018) |
| maize straw | 300 | 48.9 | 1.25 | 0.06 | 3.34 | 9.84 | wheat | - | NPK fertilizer (5, 2.5, 2 g/pot)+40.5g/pot | +100~122.73% (N uptake), +173.33~200% (P uptake), +23.38~37.66% (K uptake), +28.48~42.79% (yield) | (Song et al., 2018) |
|  | 450 | 53.81 | 1.22 | 0.06 | 6.61 | 10.47 |  |  |  |  |  |
|  | 600 | 62.9 | 1.21 | 0.06 | 5.49 | 11.37 |  |  |  |  |  |

Notes: All treatments are compared with control(without fertilizer, straw, and biochar). “-“not measured. Total carbon (TC), total nitrogen (TN), phosphorus (TP), potassium (TK) of the straw biochar used in this study. Crop height (CH), nitrogen use efficiencies (NUE), phosphorus use efficiencies (PUE), “↑” means increased, “↓” means decreased.

**References:**

Agbna, G. H. D., She, D., Liu, Z., Elshaikh, N. A., Shao, G., Timm, L. G. (2017). Effects of deficit irrigation and biochar addition on the growth, yield, and quality of tomato. *Sci. Horticulturae* 222: 90-101. doi: 10.1016/j.scienta.2017.05.004

Bai, J., Li, Y., Zhang, J., Xu, F., Bo, Q., Wang, Z., et al. (2021). Straw returning and one-time application of a mixture of controlled release and solid granular urea to reduce carbon footprint of plastic film mulching spring maize. *J. Clean. Prod.* 280, 124478. doi: 10.1016/j.jclepro.2020.124478

Bai, N., Zhang, H., Li, S., Zheng, X., Zhang, J., Zhang, H., et al. (2019). Long-term effects of straw and straw-derived biochar on soil aggregation and fungal community in a rice-wheat rotation system. *PeerJ* 6: e6171. doi: 10.7717/peerj.6171

Bai, Y., Wang, L., Lu, Y., Yang, L., Zhou, L., Ni, L., et al. (2015). Effects of long-term full straw return on yield and potassium response in wheat-maize rotation. *J. Integr. Agr.* 14: 2467-2476. doi: 10.1016/S2095-3119(15)61216-3

Bi, Q. F., Chen, Q. H., Yang, X. R., Li, H., Zheng, B. X., Zhou, W. W., et al. (2017). Effects of combined application of nitrogen fertilizer and biochar on the nitrification and ammonia oxidizers in an intensive vegetable soil. *AMB Express* **7**: 198. doi: 10.1186/s13568-017-0498-7

Chen, Y., Xin, L., Liu, J., Yuan, M., Liu, S., Jiang, W., et al. (2017). Changes in bacterial community of soil induced by long-term straw returning. *Sci. Agr.* 74: 349-356. doi: 10.1590/1678-992X-2016-0025

Chen, Y., Shi, J., Dong, J., Wu, Y., Li, C., Ye, Y., et al. (2021). Synergistic improvement of soil organic carbon storage and wheat grain zinc bioavailability by straw return in combination with Zn application on the Loess Plateau of China. *Catena* 197, 104920. doi: 10.1016/j.catena.2020.104920

Chen, Y., Fan, P., Li, L., Tian, H., Ashraf, U., Mo, Z., et al. (2020). Straw Incorporation Coupled with Deep Placement of Nitrogen Fertilizer Improved Grain Yield and Nitrogen Use Efficiency in Direct-Seeded Rice. *J. Soil Sci. Plant Nut.* 20: 2338-2347. doi: 10.1007/s42729-020-00301-2

Chen, Z., Wang, Q., Wang, H., Bao, L., Zhou, J. (2018). Crop yields and soil organic carbon fractions as influenced by straw incorporation in a rice–wheat cropping system in southeastern China. *Nutr.Cycl. Agroecosys.* 112: 61-73. doi: 10.1007/s10705-018-9929-3

Cui, Y., Meng, J., Wang, Q., Zhang, W., Cheng, X., Chen, W. (2017). Effects of straw and biochar addition on soil nitrogen, carbon, and super rice yield in cold waterlogged paddy soils of North China. *J. Integr. Agr.* 16: 1064-1074. doi: 10.1016/S2095-3119(16)61578-2

Dong, Q., Yang, Y., Yu, K., Feng, H. (2018). Effects of straw mulching and plastic film mulching on improving soil organic carbon and nitrogen fractions, crop yield and water use efficiency in the Loess Plateau, China. *Agr. Water Manage.* 201: 133-143. doi: 10.1016/j.agwat.2018.01.021

Fan, Y. F., Gao, J. L., Sun, J. Y., Liu, J., Su, Z. J., Wang, Z. J., et al. (2021). Effects of straw returning and potassium fertilizer application on root characteristics and yield of spring maize in China inner Mongolia. *Agron. J*. doi: 10.1002/agj2.20742.

Feng, W., Yang, F., Cen, R., Liu, J., Qu, Z., Miao, Q., et al. (2021). Effects of straw biochar application on soil temperature, available nitrogen and growth of corn. *J. Environ. Manage.* 277: 111331. doi: 10.1016/j.jenvman.2020.111331

Frank, Stephano. M., Geng, Y., Cao, G., Wang, L., Meng, W., Meiling, Z. (2020). Effect of Silicon Fertilizer and Straw Return on the Maize Yield and Phosphorus Efficiency in Northeast China. *Commun. Soil Sci. Plan.* 52: 116-127. doi: 10.1080/00103624.2020.1854284

Gao, F., Li, B., Ren, B., Zhao, B., Liu, P., Zhang, J. (2019). Effects of residue management strategies on greenhouse gases and yield under double cropping of winter wheat and summer maize. *Sci. Total Environ.* 687: 1138-1146. doi: 10.1016/j.scitotenv.2019.06.146

Geng, Y., Cao, G., Wang, L., Wang, S. (2019). Effects of equal chemical fertilizer substitutions with organic manure on yield, dry matter, and nitrogen uptake of spring maize and soil nitrogen distribution. *PLoS One* 14: e0219512. doi: 10.1371/journal.pone.0219512

Guan, S., Liu, S., Liu, R., Zhang, J., Ren, J., Cai, H., et al. (2019). Soil organic carbon associated with aggregate-size and density fractions in a Mollisol amended with charred and uncharred maize straw. *J. Integr. Agr.* 18: 1496-1507. doi: 10.1016/S2095-3119(19)62643-2

Guo, Z., Liu, H., Wan, S., Hua, K., Jiang, C., Wang, D., et al. (2017). Enhanced yields and soil quality in a wheat-maize rotation using buried straw mulch. *J. Sci. Food Agric.* 97: 3333-3341. doi: 10.1002/jsfa.8183

Han, Y., Ma, W., Zhou, B., Salah, A., Geng, M., Cao, C., et al. (2021). Straw return increases crop grain yields and K-use efficiency under a maize-rice cropping system. *The Crop Journal* 9: 168-180. doi: 10.1016/j.cj.2020.04.003

Han, Y., Ma, W., Zhou, B., Yang, X., Salah, A., Li, C., et al. (2020). Effects of Straw-Return Method for the Maize–Rice Rotation System on Soil Properties and Crop Yields. *Agronomy* 10, 461. doi: 10.3390/agronomy10040461

He, X., Du, Z., Wang, Y., Lu, N., Zhang, Q. (2016). Sensitivity of soil respiration to soil temperature decreased under deep biochar amended soils in temperate croplands. *Appl. Soil Ecol.* 108: 204-210. doi: 10.1016/j.apsoil.2016.08.018

Hu, Y., Sun, B., Wu, S., Feng, H., Gao, M., Zhang, B., et al. (2021). After-effects of straw and straw-derived biochar application on crop growth, yield, and soil properties in wheat (Triticum aestivum L.) -maize (Zea mays L.) rotations: A four-year field experiment. *Sci. Total Environ.* 780: 146560. doi: 10.1016/j.scitotenv.2021.146560

Huang, R., Liu, J., He, X., Xie, D., Ni, J., Xu, C., et al. (2019). Reduced mineral fertilization coupled with straw return in field mesocosm vegetable cultivation helps to coordinate greenhouse gas emissions and vegetable production. *Soil. Sediment.* 20: 1834-1845. doi: /10.1007/s11368-019-02477-2

Ji, Q., Zhao, S. X., Li, Z. H., Ma, Y. Y., Wang, X. D. (2016). Effects of Biochar-Straw on Soil Aggregation, Organic Carbon Distribution, and Wheat Growth. *Agron. J.* 108: 2129-2136. doi: 10.2134/agronj2016.02.0121

Jiang, C., Yu, W. (2019). Maize production and field CO_2_ emission under different straw return rates in Northeast China. *Plant Soil Environ.* 65: 198-204. doi: 10.17221/564/2018-PSE

Jin, Z., Chen, C., Chen, X., Jiang, F., Hopkins, I., Zhang, X., et al. (2019). Soil acidity, available phosphorus content, and optimal biochar and nitrogen fertilizer application rates: A five-year field trial in upland red soil, China. *Field Crop. Res.* 232: 77-87. doi: 10.1016/j.fcr.2018.12.013

Li, C., Ma, S., Shao, Y., Ma, S., Zhang, L. (2018a). Effects of long-term organic fertilization on soil microbiologic characteristics, yield and sustainable production of winter wheat. *J. Integr. Agr.* 17: 210-219. doi: 10.1016/S2095-3119(17)61740-4

Li, C., Xiong, Y., Qu, Z., Xu, X., Huang, Q., Huang, G. (2018b). Impact of biochar addition on soil properties and water-fertilizer productivity of tomato in semi-arid region of Inner Mongolia, China. *Geoderma* 331: 100-108. doi: 10.1016/j.geoderma.2018.06.014

Li, F., Liang, X., Zhang, H., Tian, G. (2019a). The influence of no-till coupled with straw return on soil phosphorus speciation in a two-year rice-fallow practice. *Soil Till. Res.* 195, 104389. doi: 10.1016/j.still.2019.104389

Li, J., Li, H., Zhang, Q., Shao, H., Gao, C., Zhang, X. (2019b). Effects of fertilization and straw return methods on the soil carbon pool and CO_2_ emission in a reclaimed mine spoil in Shanxi Province, China. *Soil Till. Res.* 195, 104361. doi: 10.1016/j.still.2019.104361

Li, J., Wu, X., Gebremikael, M. T., Wu, H., Cai, D., Wang, B., et al. (2018c). Response of soil organic carbon fractions, microbial community composition and carbon mineralization to high-input fertilizer practices under an intensive agricultural system. *PLoS One* 13: e0195144. doi: 10.1371/journal.pone.0195144

Li, S., Wang, S., Shangguan, Z. (2019d). Combined biochar and nitrogen fertilization at appropriate rates could balance the leaching and availability of soil inorganic nitrogen. *Agr. Ecosyst. Environ.* 276: 21-30. doi: 10.1016/j.agee.2019.02.013

Li, S., Li, X., Zhu, W., Chen, J., Tian, X., Shi, J. (2019c). Does Straw Return Strategy Influence Soil Carbon Sequestration and Labile Fractions? *Agron. J.* 111: 897-906. doi: 10.2134/agronj2018.08.0484

Li, X., Song, Y., Wang, F., Bian, Y., Jiang, X. (2019e). Combined effects of maize straw biochar and oxalic acid on the dissipation of polycyclic aromatic hydrocarbons and microbial community structures in soil: A mechanistic study. *J. Hazard. Mater.* 364: 325-331. doi: 10.1016/j.jhazmat.2018.10.041

Li, X., Liang, Z., Li, Y., Zhu, Y., Tian, X., Shi, J., et al. (2021a). Short‐term effects of combined organic amendments on soil organic carbon sequestration in a rain‐fed winter wheat system. *Agron. J.* 113: 2150-2164. doi: 10.1002/agj2.20624

Li, Y., Yang, R., Gao, R., Wei, H., Chen, A., Li, Y. (2015). Effects of long-term phosphorus fertilization and straw incorporation on phosphorus fractions in subtropical paddy soil. *J. Integr. Agr.* 14: 365-373. doi: 10.1016/S2095-3119(13)60684-X

Li, Y., Chen, J., Feng, H., Dong, Q., Siddique, K. H. M. (2021b). Responses of canopy characteristics and water use efficiency to ammoniated straw incorporation for summer maize (Zea mays L.) in the Loess Plateau, China. *Agr. Water Manage.* 254, 106948. doi: 10.1016/j.agwat.2021.106948

Lin, X. W., Xie, Z. B., Zheng, J. Y., Liu, Q., Bei, Q. C. Zhu, J. G. (2015). Effects of biochar application on greenhouse gas emissions, carbon sequestration and crop growth in coastal saline soil. *Eur. J. Soil Sci.* 66: 329-338. doi: 10.1111/ejss.12225

Liu, D., Feng, Z., Zhu, H., Yu, L., Yang, K., Yu, S., et al. (2020a). Effects of Corn Straw Biochar Application on Soybean Growth and Alkaline Soil Properties. BioResources 15, 1463-1481. doi: 10.15376/BIORES.15.1.1463-1481

Liu, J., Jiang, B., Shen, J., Zhu, X., Yi, W., Li, Y., et al. (2021). Contrasting effects of straw and straw-derived biochar applications on soil carbon accumulation and nitrogen use efficiency in double-rice cropping systems. *Agr. Ecosyst. Environ.* 311, 107286. doi: 10.1016/j.agee.2020.107286

Liu, X., Zhang, D., Li, H., Qi, X., Gao, Y., Zhang, Y., et al. (2020b). Soil nematode community and crop productivity in response to 5-year biochar and manure addition to yellow cinnamon soil. *BMC Ecol.* 20, 39. doi: 10.1186/s12898-020-00304-8

Liu, Y., Zhu, J., Gao, W., Guo, Z., Xue, C., Pang, J., et al. (2019). Effects of biochar amendment on bacterial and fungal communities in the reclaimed soil from a mining subsidence area. *Environ. Sci. Pollut Res. Int.* 26: 34368-34376. doi: 10.1007/s11356-019-06567-z

Luo, S., Gao, Q., Wang, S., Tian, L., Zhou, Q., Li, X., et al. (2020). Long-term fertilization and residue return affect soil stoichiometry characteristics and labile soil organic matter fractions. *Pedosphere* 30: 703-713. doi: 10.1016/S1002-0160(20)60031-5

Lv, Y., Wang, Y., Wang, L., Zhu, P. (2019). Straw Return with Reduced Nitrogen Fertilizer Maintained Maize High Yield in Northeast China. *Agronomy* 9, 229. doi:10.3390/agronomy9050229

Ma, S., Kan, Z., Qi, J., Zhang, H. (2020). Effects of Straw Return Mode on Soil Aggregates and Associated Carbon in the North China Plain. *Agronomy* 10, 61. doi:10.3390/agronomy10010061

Ma, Z., Xie, Y., Zhu, L., Cheng, L., Xiao, X., Zhou, C., et al. (2017). Which of soil microbes is in positive correlation to yields of maize (Zea mays L.)? *Plant Soil Environ.* 63: 574-580. doi: 10.17221/590/2017-PSE

Ndzelu, B. S., Dou, S., Zhang, X. (2021). Corn straw return can increase labile soil organic carbon fractions and improve water-stable aggregates in Haplic Cambisol. *J. Arid Land* 12: 1018-1030. doi: 10.1007/s40333-020-0024-7

Qiao, Y., Miao, S., Zhong, X., Zhao, H., Pan, S. (2020). The greatest potential benefit of biochar return on bacterial community structure among three maize-straw products after eight-year field experiment in Mollisols. *Appl. Soil Ecol.* 147. doi: 10.1016/j.apsoil.2019.103432

Shaaban, M., Wu, Y., Peng, Q., Wu, L., VanZwieten, L., Khalid, M. S., et al. (2018). The interactive effects of dolomite application and straw incorporation on soil N_2_O emissions. *Eur. J. Soil Sci.* 69: 502-511. doi: 10.1111/ejss.12541

Shi, Y., Liu, X., Zhang, Q. (2019a). Effects of combined biochar and organic fertilizer on nitrous oxide fluxes and the related nitrifier and denitrifier communities in a saline-alkali soil. *Sci. Total Environ.* 686: 199-211. doi: 10.1016/j.scitotenv.2019.05.394

Shi, Y., Liu, X., Zhang, Q., Gao, P., Ren, J. (2019b). Biochar and organic fertilizer changed the ammonia-oxidizing bacteria and archaea community structure of saline–alkali soil in the North China Plain. *J. Soil. Sediment.* 20: 12-23. doi: 10.1007/s11368-019-02364-w

Si, L., Xie, Y., Ma, Q., Wu, L. (2018). The Short-Term Effects of Rice Straw Biochar, Nitrogen and Phosphorus Fertilizer on Rice Yield and Soil Properties in a Cold Waterlogged Paddy Field. *Sustainability* 10, 537. doi:10.3390/su10020537

Song, A., Li, Z., Liao, Y., Liang, Y. C., Wang, E. Z., Wang, S., et al. (2021). Soil bacterial communities interact with silicon fraction transformation and promote rice yield after long-term straw return. *Soil Ecol. Lett*. doi: 10.1007/s42832-021-0076-4

Song, D., Tang, J., Xi, X., Zhang, S., Liang, G., Zhou, W., et al. (2018). Responses of soil nutrients and microbial activities to additions of maize straw biochar and chemical fertilization in a calcareous soil. *Eur. J. Soil Biol.* 84: 1-10. doi: 10.1016/j.ejsobi.2017.11.003

Sui, Y., Gao, J., Liu, C., Zhang, W., Lan, Y., Li, S., et al. (2016). Interactive effects of straw-derived biochar and N fertilization on soil C storage and rice productivity in rice paddies of Northeast China. *Sci. Total Environ.* 544: 203-210. doi: 10.1016/j.scitotenv.2015.11.079

Tan, G., Wang, H., Xu, N., Junaid, M., Liu, H., Zhai, L. (2021). Effects of biochar application with fertilizer on soil microbial biomass and greenhouse gas emissions in a peanut cropping system. *Environ. Technol.* 42: 9-19. doi: 10.1080/09593330.2019.1620344

Tian, P., Lian, H., Wang, Z., Jiang, Y., Li, C., Sui, P., et al. (2020). Effects of Deep and Shallow Tillage with Straw Incorporation on Soil Organic Carbon, Total Nitrogen and Enzyme Activities in Northeast China. *Sustainability* 12, 8679. doi:10.3390/su12208679

Wang, W., Lai, D., Abid, A., Neogi, S., Xu, X., Wang, C. (2018). Effects of Steel Slag and Biochar Incorporation on Active Soil Organic Carbon Pools in a Subtropical Paddy Field. *Agronomy* 8, 135. doi:10.3390/agronomy8080135

Wang, W., Akhtar, K., Ren, G., Yang, G., Feng, Y., Yuan, L. (2019). Impact of straw management on seasonal soil carbon dioxide emissions, soil water content, and temperature in a semi-arid region of China. *Sci. Total Environ.* 652: 471-482. doi: 10.1016/j.scitotenv.2018.10.207

Wang, X., Zhou, W., Liang, G., Song, D., Zhang, X. (2015b). Characteristics of maize biochar with different pyrolysis temperatures and its effects on organic carbon, nitrogen and enzymatic activities after addition to fluvo-aquic soil. *Sci. Total Environ.* 538: 137-144. doi: 10.1016/j.scitotenv.2015.08.026

Wang, X., Song, D., Liang, G., Zhang, Q., Ai, C., Zhou, W. (2015a). Maize biochar addition rate influences soil enzyme activity and microbial community composition in a fluvo-aquic soil. *Appl. Soil Ecol.* 96: 265-272. doi: 10.1016/j.apsoil.2015.08.018

Xiao, Q., Zhu, L. X., Zhang, H. P., Li, X. Y., Shen, Y. F., Li, S. Q. (2016). Soil amendment with biochar increases maize yields in a semi-arid region by improving soil quality and root growth. *Crop Pasture Sci.* 67, 495-507. doi: 10.1071/CP15351

Xie, W. Y., Yuan, S. T., Xu, M. G., Yang, X. P., Shen, Q. R., Zhang, W. W.,et al. (2018). Long-term effects of manure and chemical fertilizers on soil antibiotic resistome. *Soil Biol. Biochem.* 122: 111-119. doi: 10.1016/j.soilbio.2018.04.009

Xie, Y., Dong, C., Chen, Z., Liu, Y. J., Zhang, Y. Y., Guo, P. X., et al. (2021). Successive biochar amendment affected crop yield by regulating soil nitrogen functional microbes in wheat-maize rotation farmland. *Environ. Res.* 194: 110671. doi: 10.1016/j.envres.2020.110671

Xiu, L., Zhang, W., Sun, Y., Wu, D., Meng, J., Chen, W. (2019). Effects of biochar and straw returning on the key cultivation limitations of Albic soil and soybean growth over 2 years. *Catena* 173: 481-493. doi: 10.1016/j.catena.2018.10.041

Xu, C. Y., Hosseini-Bai, S., Hao, Y., Rachaputi, R. C., Wang, H., Xu, Z., et al. (2015). Effect of biochar amendment on yield and photosynthesis of peanut on two types of soils. *Environ. Sci. Pollut. Res. Int* 22: 6112-6125. doi: 10.1007/s11356-014-3820-9

Xu, N., Tan, G., Wang, H., Gai, X. (2016a). Effect of biochar additions to soil on nitrogen leaching, microbial biomass and bacterial community structure. *Eur. J. Soil Biol.* 74: 1-8. doi: 10.1016/j.ejsobi.2016.02.004

Xu, P., Sun, C. X., Ye, X. Z., Xiao, W. D., Zhang, Q., Wang, Q. (2016b). The effect of biochar and crop straws on heavy metal bioavailability and plant accumulation in a Cd and Pb polluted soil. *Ecotoxicol Environ. Saf.* 132: 94-100. doi: 10.1016/j.ecoenv.2016.05.031

Xu, X., Pang, D., Chen, J., Luo, Y., Zheng, M., Yin, Y., et al. (2018). Straw return accompany with low nitrogen moderately promoted deep root. *Field Crop. Res.* 221: 71-80. doi: 10.1016/j.fcr.2018.02.009

Yan, C., Yan, S. S., Jia, T. Y., Dong, S. K., Ma, C. M., Gong, Z. P. (2019a). Decomposition characteristics of rice straw returned to the soil in northeast China. *Nutr. Cycl. Agroecosys.* 114: 211-224. doi: 10.1007/s10705-019-09999-8

Yan, Q., Dong, F., Li, J., Duan, Z., Yang, F., Li, X., et al. (2019b). Effects of maize straw-derived biochar application on soil temperature, water conditions and growth of winter wheat. *Eur. J. Soil Sci.* 70: 1280-1289. doi: 10.1111/ejss.12863

Yang, J., Gao, W., Ren, S. (2015). Long-term effects of combined application of chemical nitrogen with organic materials on crop yields, soil organic carbon and total nitrogen in fluvo-aquic soil. *Soil Till. Res.* 151: 67-74. doi: 10.1016/j.still.2015.03.008

Yang, L., Bai, J., Zeng, N., Zhou, X., Liao, Y., Lu, Y., et al. (2019). Diazotroph abundance and community structure are reshaped by straw return and mineral fertilizer in rice-rice-green manure rotation. *Appl. Soil Ecol.* 136: 11-20. doi: 10.1016/j.apsoil.2018.12.015

Yang, S., Xiao, Y., Xu, J., Liu, X. (2018). Effect of straw return on soil respiration and NEE of paddy fields under water-saving irrigation. *PLoS One* 13: e0204597. doi: 10.1371/journal.pone.0204597

Yang, X., Liu, H., Mao, X., Deng, J., Haefele, S. M. (2020). Non‐flooding rice yield response to straw biochar and controlled‐release fertilizer. *Agron. J.* 112: 4799-4809. doi: 10.1002/agj2.20430.

Yao, Z., Yan, G., Zheng, X., Wang, R., Liu, C., Butterbach-Bahl, K. (2017). Straw return reduces yield-scaled N_2_O plus NO emissions from annual winter wheat-based cropping systems in the North China Plain. *Sci. Total Environ.* 590-591: 174-185. doi: 10.1016/j.scitotenv.2017.02.194

Yin, W., Guo, Y., Hu, F., Fan, Z., Feng, F., Zhao, C., et al. (2018). Wheat-Maize Intercropping With Reduced Tillage and Straw Retention: A Step Towards Enhancing Economic and Environmental Benefits in Arid Areas. *Front. Plant Sci.* 9: 1328. doi: 10.3389/fpls.2018.01328

Yu, L., Lu, X., He, Y., Brookes, P. C., Liao, H., Xu, J. (2016). Combined biochar and nitrogen fertilizer reduces soil acidity and promotes nutrient use efficiency by soybean crop. *J. Soil. Sediment.* 17: 599-610. doi: 10.1007/s11368-016-1447-9

Yu, L., Yu, M., Lu, X., Tang, C., Liu, X., Brookes, P. C. et al. (2018). Combined application of biochar and nitrogen fertilizer benefits nitrogen retention in the rhizosphere of soybean by increasing microbial biomass but not altering microbial community structure. *Sci. Total Environ.* 640-641: 1221-1230. doi: 10.1016/j.scitotenv.2018.06.018

Yuan, G., Huan, W., Song, H., Lu, D., Chen, X., Wang, H., et al. (2021). Effects of straw incorporation and potassium fertilizer on crop yields, soil organic carbon, and active carbon in the rice–wheat system. *Soil Till. Res.* 209, 104958. doi: 10.1016/j.still.2021.104958

Zhai L, CaiJi Z, Liu J, Wang H, Ren T, Gai X, Xi B & Liu H (2015). Short-term effects of maize residue biochar on phosphorus availability in two soils with different phosphorus sorption capacities. *Biol. Fert. Soils* 51: 113-122. doi: 10.1007/s00374-014-0954-3

Zhang, A., Cheng, G., Hussain, Q., Zhang, M., Feng, H., Dyck, M., et al. (2017). Contrasting effects of straw and straw–derived biochar application on net global warming potential in the Loess Plateau of China. *Field Crop. Res.* 205: 45-54. doi: 10.1016/j.fcr.2017.02.006

Zhang, D., Pan, G., Wu, G., Kibue, G. W., Li, L. Q., Zhang, X. H., et al. (2016a). Biochar helps enhance maize productivity and reduce greenhouse gas emissions under balanced fertilization in a rainfed low fertility inceptisol. *Chemosphere* 142: 106-113. doi: 10.1016/j.chemosphere.2015.04.088

Zhang, J., Huang, Y., Lin, J., Chen, X., Li, C., Zhang, J. (2019). Biochar applied to consolidated land increased the quality of an acid surface soil and tobacco crop in Southern China. *J. Soil. Sediment.* 20: 3091-3102. doi: 10.1007/s11368-019-02531-z

Zhang, M., Geng, Y., Cao, G., Wang, L., Wang, M., Stephano, M. F. (2020a). Magnesium accumulation, partitioning and remobilization in spring maize (Zea mays L.) under magnesium supply with straw return in northeast China. *J. Sci. Food Agric.* 100: 2568-2578. doi: 10.1002/jsfa.10282

Zhang, M., Geng, Y., Cao, G., Zou, X., Qi, X., Stephano, M. F. (2020b). Effect of magnesium fertilizer combined with straw return on nitrogen use efficiency. *Agron. J.* 113: 345-357. doi: 10.1002/agj2.20483

Zhang, Q., Song, Y., Wu, Z., Yan, X., Gunina, A., Kuzyakov, Y., et al. (2020c). Effects of six-year biochar amendment on soil aggregation, crop growth, and nitrogen and phosphorus use efficiencies in a rice-wheat rotation. *J. Clean. Prod.* 242. doi: 10.1016/j.jclepro.2019.118435

Zhang, Y., Liu, Y., Zhang, G., Guo, X., Sun, Z., Li, T. (2018). The Effects of Rice Straw and Biochar Applications on the Microbial Community in a Soil with a History of Continuous Tomato Planting History. *Agronomy* 8, 65. doi:10.3390/agronomy8050065

Zhang, Y., Li, C., Wang, Y., Hu, Y., Christie, P., Zhang, J., et al. (2016b). Maize yield and soil fertility with combined use of compost and inorganic fertilizers on a calcareous soil on the North China Plain. *Soil Till. Res.* 155: 85-94. doi: 10.1016/j.still.2015.08.006

Zhao, S., Li, K., Zhou, W., Qiu, S., Huang, S., He, P. (2016a). Changes in soil microbial community, enzyme activities and organic matter fractions under long-term straw return in north-central China. *Agr. Ecosyst. Environ.* 216: 82-88. doi: 10.1016/j.agee.2015.09.028

Zhao, S., Qiu, S., Xu, X., Ciampitti, I. A., Zhang, S., He, P. (2019a). Change in straw decomposition rate and soil microbial community composition after straw addition in different long-term fertilization soils. *Appl. Soil Ecol.* 138: 123-133. doi: 10.1016/j.apsoil.2019.02.018

Zhao, W. R., Li, J. Y., Deng, K. Y., Shi, R. Y., Jiang, J., Hong, Z. N., et al. (2020). Effects of crop straw biochars on aluminum species in soil solution as related with the growth and yield of canola (*Brassica napus* L.) in an acidic Ultisol under field condition. *Environ. Sci. Pollut. Res. Int.* 27: 30178-30189. doi: 10.1007/s11356-020-09330-x

Zhao, X., Yuan, G., Wang, H., Lu, D., Chen, X., Zhou, J. (2019b). Effects of Full Straw Incorporation on Soil Fertility and Crop Yield in Rice-Wheat Rotation for Silty Clay Loamy Cropland. *Agronomy* 9, 133. doi: 10.3390/agronomy9030133

Zhao, Y., Zhang, Y., Liu, X., He, X., Shi, X. (2016b). Carbon sequestration dynamic, trend and efficiency as affected by 22-year fertilization under a rice-wheat cropping system. *J. Plant Nutr. Soil Sc.* 179: 652-660. doi: 10.1002/jpln.201500602

Zhou, H., Fang, H., Zhang, Q., Wang, Q., Chen, C., Mooney, S. J., et al. (2018). Biochar enhances soil hydraulic function but not soil aggregation in a sandy loam. *Eur. J. Soil Sci.* 70: 291-300. doi: 10.1111/ejss.12732

Zhu, L., Hu, N., Zhang, Z., Xu, J., Tao, B., Meng, Y. (2015a). Short-term responses of soil organic carbon and carbon pool management index to different annual straw return rates in a rice–wheat cropping system. *Catena* 135: 283-289. doi: 10.1016/j.catena.2015.08.008

Zhu, Q., Peng, X., Huang, T. (2015b). Contrasted Effects of Biochar on Maize Growth and N Use Efficiency Depending on Soil Conditions. *Int. Agrophys.* 29: 257-266. doi: 10.1515/intag-2015-0023
